# Biological activities and safety assessment of *Teleogryllus mitratus* extracts for skin delivery via nanoemulsion-based systems

**DOI:** 10.1016/j.ijpx.2026.100542

**Published:** 2026-04-10

**Authors:** Jirasit Inthorn, Pratthana Chomchalao, Saranya Juntrapirom, Watchara Kanjanakawinkul, Andrea Heinz, Anette Müllertz, Thomas Rades, Wantida Chaiyana

**Affiliations:** a Department of Pharmaceutical Sciences, Faculty of Pharmacy, Chiang Mai University, Chiang Mai 50200, Thailand; b College of Medicine and Public Health, Ubon Ratchathani University, Ubon Ratchathani 34190, Thailand; c Chulabhorn Royal Pharmaceutical Manufacturing Facilities by Chulabhorn Royal Academy, Chon Buri 20180, Thailand; d Department of Pharmacy, LEO Foundation Center for Cutaneous Drug Delivery, University of Copenhagen, 2100 Copenhagen, Denmark; e Bioneer: FARMA, Department of Pharmacy, University of Copenhagen, Universitetsparken 4, Copenhagen 2100, Denmark; f Department of Pharmacy, Faculty of Health and Medical Sciences, University of Copenhagen, Universi-tetsparken 2, 2100 Copenhagen, Denmark; g Center of Excellence in Pharmaceutical Nanotechnology, Faculty of Pharmacy, Chiang Mai University, Chiang Mai 50200, Thailand; h Research Center of Deep Technology in Beekeeping and Bee Products for Sustainable Development Goals (SMART BEE SDGs), Chiang Mai University, Chiang Mai 50200, Thailand; i Multidisciplinary and Interdisciplinary School, Chiang Mai University, Chiang Mai 50200, Thailand

**Keywords:** Ground cricket, Enzyme-assisted extraction, Green extraction, Anti-aging, Cosmeceutical, Nanotechnology, Nanodelivery system

## Abstract

This study aimed to investigate the cosmeceutical effects of cricket extracts and assess their delivery through a nanoemulsion system. Different species of crickets (*Gryllus bimaculatus*, *Teleogryllus mitratus*, and *Acheta domesticus*) were freeze-dried, defatted, and extracted using aqueous, enzyme-assisted, and protein isolation methods. Extracts were evaluated for total protein content, amino acid composition, anti-tyrosinase, and anti-hyaluronidase activities. The most bioactive extract was incorporated into an oil-in-water nanoemulsion, which was characterized for droplet size, polydispersity index (PDI), zeta potential, morphology, entrapment efficiency (EE), release profile, in vitro permeation and retention. The Subtilisin A-assisted extract of *T. mitratus* (TMS) contained the highest protein content (76.0 ± 1.1% *w*/w) and potent bioactivity, with tyrosinase IC_50_ values of 65.4 ± 6.9 μg/mL (L-tyrosine) and 395.9 ± 77.2 μg/mL (L-DOPA), and an anti-hyaluronidase IC_50_ of 12.5 ± 3.5 μg/mL. Nanoemulsion incorporation achieved 46.36 ± 1.69% EE and 0.20 ± 0.01% loading capacity, with nanoscale droplet sizes (288.3 ± 11.3 nm) and high stability (PDI < 0.3; zeta potential < −30 mV). Therefore, the TMS-loaded nanoemulsion provided multifunctional cosmeceutical benefits.

## Highlights

• Enzyme-assisted *T. mitratus* extract (TMS) inhibited tyrosinase and hyaluronidase.
• The nanoemulsion with a droplet size of 288 nm achieved 46% entrapment of TMS.
• The nanoemulsion improved skin delivery and retention of TMS.

## Graphical abstract

**Figure:**
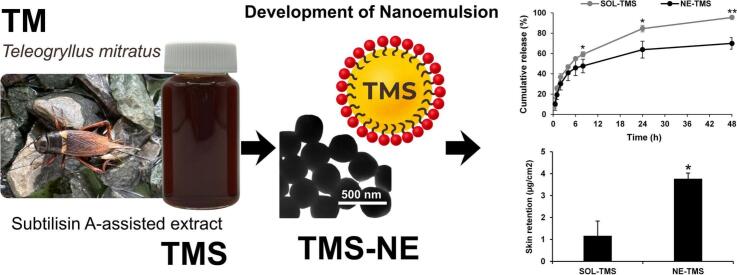

## 1 Introduction

Insects (Arthropoda: Insecta) are the most diverse group of organisms on earth, encompassing over a million documented species (Engel, 2015; Yi et al., 2011). Beyond their ecological significance, edible insects have garnered growing interest as a sustainable and nutritious food source, offering high protein content, essential amino acids, and bioactive compounds, while exerting a remarkably low environmental impact compared to conventional livestock farming (Moruzzo et al., 2021). Recent research has highlighted that insect-derived bioactive compounds, particularly proteins and peptides, possess multifunctional properties that extend beyond nutrition, including antioxidant, anti-inflammatory, and antimicrobial activities (Zielinska et al., 2017; Zielinska et al., 2018; Ferrazzano et al., 2023). Crickets are among the group of edible insects, valued for their nutritional qualities (Kim et al., 2020). Specifically, *Gryllus bimaculatus*, *Teleogryllus mitratus*, and *Acheta domesticus* are widely cultivated edible species in Thailand, recognized for their nutritional richness (Ruang-Rit et al., 2025; Mitchaothai et al., 2024; van Huis, 2020). While these species are traditionally used for human consumption, recent research suggests their potential application in cosmeceuticals, offering antioxidant and anti-aging benefits (Chaiyana et al., 2024; Yeerong et al., 2024b; Yeerong et al., 2021).

Bioactive peptides are increasingly recognized as valuable cosmetic ingredients due to their diverse biological activities, including antioxidant, anti-aging, anti-inflammatory, and antimicrobial effects (Ngoc et al., 2023). These peptides can function through multiple mechanisms, such as radical scavenging, modulation of inflammatory pathways, and inhibition of enzymes related to skin aging (e.g., collagenase, elastase, and tyrosinase) (Aguilar-Toalá et al., 2019). Peptides and proteins derived from edible insects have been proposed as functional active components with potential as natural cosmetic ingredients (Kim, 2017; Kim et al., 2023). Kim (2017) reported their possible roles in wrinkle improvement and skin health, including those obtained from silk moth. Besides, the insect-derived peptides have been explored as functional cosmetic ingredients due to their moisturizing and UV-protective properties, attributed to their high protein content (Kim et al., 2023). Despite a growing interest in natural and sustainable cosmetic ingredients, crickets remain largely unexplored in cosmetic formulation, highlighting a novel opportunity for the development of innovative, protein-based cosmeceutical products.

Despite the potential of crickets in cosmetics, the direct use of crude cricket extracts is often limited by poor skin penetration and low stability of their bioactive proteins and peptides (Yeerong et al., 2025). These challenges highlight the need for innovative delivery strategies to maximize the functional benefits of cricket-derived bioactive compounds. With the growing consumer demand for more effective products, cosmeceuticals, defined as products with measurable biological activity in the skin, represent the fastest-growing segment of the personal care industry (Yukuyama et al., 2016; Lohani et al., 2014). Achieving a desired cosmetic effect requires sufficient penetration of active ingredients into the skin layer, while ensuring that they are not systemically absorbed following topical application (Pardeike et al., 2009). Considering the complex structure and barrier function of the skin, lipid-based formulations are widely regarded as the most suitable vehicles for the topical delivery of active compounds (Souto and Muller, 2008). Emulsion systems at the nanoscale, coupled with carefully optimized processing techniques, provide an effective strategy to improve skin penetration and maximize the efficacy of the actives (Yukuyama et al., 2016). Nanoemulsion-based delivery systems have emerged as effective carriers to enhance the penetration, stability, and controlled release of bioactive compounds in cosmeceutical formulations (Yukuyama et al., 2016; Harwansh et al., 2019; Ashaolu, 2021).

Nanoemulsions offer several advantages over conventional emulsions, including small droplet size for increased surface area and enhanced absorption, efficient solubilization of lipophilic compounds, chemical stabilization of sensitive actives, and generally low toxicity and irritation (Jaiswal et al., 2015; Kim et al., 2001). In contrast, for hydrophilic compounds such as proteins, nanoemulsions primarily function as stabilizing and protective delivery system rather than as solubilization vehicles. Previous studies have reported that flexible proteins such as sodium caseinate and rigid globular soy protein isolates can adsorb at the oil–water interface, forming a protective interfacial layer around the droplets, while a small fraction of protein molecules remains freely dispersed in the aqueous phase (Ji et al., 2015). The stability of these protein-stabilized nanoemulsions was mainly attributed to electrostatic repulsion between droplets, along with additional steric stabilization provided by the adsorbed protein layers (Ji et al., 2015). Therefore, these properties make nanoemulsions particularly suitable for protein delivery, as they can protect proteins from degradation, improve their bioavailability, and facilitate efficient penetration into the skin.

The present study aimed to investigate the cosmeceutical potential of cricket-derived protein extracts from different species and to develop a nanoemulsion system for enhancing their stability, bioactivity, and skin delivery.

## 2 Materials and methods

### 2.1 Chemical materials

Subtilisin A from *Bacillus licheniformis* (2.4 Anson units/g, E.C. 3.4.21.62, Sigma P4860), papain, protease from *Carica papaya* (10 units/mg, E.C. 3.4.22.2, Sigma P4762), trypsin from porcine pancreas (13,000–20,000 N-α-benzoyl-L-arginine ethyl ester hydrochloride units/mg, E.C. 3.4.21.4, Sigma T0303), tyrosinase from mushroom (1000 unit/mg solid, E.C. 1.14.18.1, Sigma T3824), hyaluronidase from bovine testes (400–1000 units/mg solid, E.C. 253–464-3, Sigma H3506), sodium lauryl sulfate (SLS), sodium chloride (NaCl), and ethylenediaminetetraacetic acid (EDTA) were purchased from Sigma-Aldrich (St Louis, MO, USA). 3,4-Dihydroxyphenylalanine (L-DOPA) was purchased from Loba Chemie (Mumbai, India). The bicinchoninic acid (BCA) protein assay kit was purchased from EMD Millipore Corp. (Darmstadt, Germany). Kojic acid, oleanolic acid, linoleic acid, bovine serum albumin (BSA), and hyaluronic acid sodium salt from *Streptococcus pyrogenes* were purchased from Merck KGaA (Darmstadt, Germany). Absolute ethanol, methanol, deionized (DI) water, and sodium hydroxide (NaOH) were analytical grade and purchased from RCI Labscan Ltd. (Bangkok, Thailand).

### 2.2 Cricket materials

Different types of frozen crickets, including field crickets (*Gryllus bimaculatus*), ground crickets (*Teleogryllus mitratus*), and house crickets (*Acheta domesticus*), which are available as edible insects for cooking, were purchased from a local farm in Chiang Mai, Thailand. The frozen crickets were freeze-dried using a freeze dryer (Kinetic Engineering Co., Ltd., Bangkok, Thailand) for 24 h until completely dry. The dried crickets were then defatted through a mechanical process using a cold-pressing machine (FEA-100SS-M-H-TC, Energy Friend Ltd. Part., Chiang Mai, Thailand). After fat removal, the resulting cricket residue was ground into powder, followed by treatment with absolute ethanol, according to a previous study by Inthorn et al. (2024). The defatted cricket powder was dried at ambient temperature and stored in a sealed aluminum foil bag until further experimentation.

### 2.3 Extraction techniques for bioactive compounds from crickets

#### 2.3.1 Enzyme-assisted extraction

The defatted *T. mitratus* powder sample (30 g) was enzymatically extracted using different proteases, including subtilisin A, papain, and trypsin. Various enzymes were compared based on their distinct proteolytic specificities. Subtilisin A is a broad-spectrum bacterial serine protease (Leroy et al., 2008), papain is a cysteine protease (Yazawa and Numata, 2014), whereas trypsin is a serine protease that specifically cleaves peptide bonds at the C-terminal side of lysine and arginine residues (Olsen et al., 2004). Firstly, the cricket powder was dispersed in DI water and pasteurized at 90 °C for 15 min. The enzyme-assisted extraction conditions for each enzyme were based on the optimal parameters specified in the supplier's technical data, as well as optimized conditions from previous studies. Following pasteurization, the pH of the dispersion was adjusted accordingly, and extraction was performed for 4 h under specific conditions, including at pH 8.0 and 60 °C with an enzyme-to-substrate weight ratio of 10:100 for subtilisin A (Hall et al., 2018), at pH 7.0 and 60 °C with a ratio of 1:200 for papain (Pali-Scholl et al., 2019; Meinlschmidt et al., 2016), and at pH 8.5 and 37 °C with a ratio of 1:100 for trypsin (Leni et al., 2020). All extraction processes were conducted under light-protected conditions. Finally, the resulting extracts were pasteurized again to inactivate the enzymes and then allowed to cool to room temperature. The extracts were subsequently centrifuged at 3200 ×*g* for 20 min at 4 °C. The supernatant was collected, and its pH was adjusted to 7.0 before lyophilization (CHRIST Beta 2–8 LDplus freeze dryer, Martin Christ Gefriertrocknungsanlagen GmbH, Osterode am Harz, Germany). The dried enzyme-assisted extracts, including those from subtilisin A (TMS), papain (TMP), and trypsin (TMT), were kept in a sealed aluminum foil bag and stored at −20 °C until further experimentation.

#### 2.3.2 Protein isolation

The defatted *T. mitratus* cricket powder sample (30 g) was dispersed in 300 mL of DI water, and the pH was adjusted to 12.0 by adding a 2.5 M NaOH solution. Extraction was carried out under continuous agitation using a magnetic stirrer (IKA® C-MAG HS7, IKA Werke GmbH & Co. KG, Staufen, Germany) set at 500 rpm for 4 h at room temperature. The resulting mixture was centrifuged at 3200 ×*g* for 20 min at 4 °C using an ultracentrifuge (MPW-352R, MPW MED. INSTRUMENTS, Warsaw, Poland). The supernatant was collected and adjusted to a lower pH using a 2.5 M hydrochloric acid solution. After reaching the isoelectric point of pH 4.0 (Mba et al., 2021; Choi et al., 2017; Quinteros et al., 2022), the resulting mixture was further stirred for an additional 1 h at room temperature and subsequently subjected to centrifugation at 3200 ×*g* for 20 min at 4 °C. The insoluble protein was collected and redispersed in DI water. After that, the pH was adjusted to pH 7.0 before lyophilization (CHRIST Beta 2–8 LDplus freeze dryer, Martin Christ Gefriertrocknungsanlagen GmbH, Osterode am Harz, Germany). The dried protein isolate (TMI) was kept in a sealed aluminum foil bag and stored at −20 °C until further experimentation.

### 2.4 Protein analysis of cricket extracts

#### 2.4.1 Determination of total protein content by bicinchoninic acid (BCA) assay

The total protein content of each cricket extract was determined using a BCA assay, following the method described by Mba et al. (2021). Briefly, 25 μL of each sample solution was mixed with 200 μL of BCA working reagent and incubated at 37 °C for 30 min. The absorbance was then measured at 562 nm using a microplate reader (CLARIOstar PLUS, BMG Labtech, Ortenberg, Germany). The absorbance values of each sample were used to calculate the total protein content, expressed as g BSA/g extract, based on the equation of the standard curve plotted from absorbance values against BSA concentration. All experiments were performed in triplicate.

#### 2.4.2 Determination of total protein content by nitrogen analyzer

The nitrogen content of each cricket extract was analyzed using a nitrogen analyzer according to the Dumas combustion method (Dumatherm N Pro, Gerhardt GmbH & Co. KG, Königswinter, Germany). In brief, 20 mg of each cricket extract was placed on the sample feeding plate and combusted in a reactor at 1030 °C using helium, nitrogen, and oxygen as carrier gases. EDTA was used as a standard substance to calibrate the nitrogen content (Choi et al., 2017; Quinteros et al., 2022). The resulting nitrogen weight was recorded and converted to the protein content using a standard protein-to‑nitrogen conversion factor of 6.25 (Choi et al., 2017). The total protein content of each extract was calculated using the following equation: Total protein content (%) = [(*A/B*) × 100] × 6.25, where *A* refers to the weight of nitrogen and *B* refers to the weight of the cricket extract. All experiments were performed in triplicate.

### 2.5 Determination of cosmeceutical effects of cricket extracts

#### 2.5.1 Anti-tyrosinase activities

The anti-tyrosinase activity of each cricket extract was determined according to the methods of Kim et al. (2012) and Deori et al. (2014). L-tyrosine and L-DOPA were used as substrates for the assay and prepared at a concentration of 9 mM in PBS pH 6.8. Briefly, a tyrosinase solution was prepared at a concentration of 125 units/mL in 50 mM PBS pH 6.8. Subsequently, 50 μL of each cricket extract was incubated with 40 μL of tyrosinase enzyme solution at ambient temperature for 10 min. Following this, 110 μL of the substrate solution was added, and the resulting mixture was further incubated at ambient temperature for 30 min. The absorbance was measured at 492 nm using a microplate reader (CLARIOstar PLUS, BMG Labtech, Ortenberg, Germany). The tyrosinase inhibition of each cricket extract was calculated using the following equation: Tyrosinase inhibition (%) = [(*A–B*)/*A*] × 100, where *A* refers to the absorbance resulting from the reaction without the cricket extracts, and *B* refers to the absorbance of the reaction resulting from the cricket extracts. Kojic acid served as a positive control. The IC_50_ values were calculated using GraphPad Prism (version 10.0, San Diego, CA, USA). All experiments were performed in triplicate.

#### 2.5.2 Anti-hyaluronidase activity determination

The anti-hyaluronidase activity of each cricket extract was determined according to the methods of Bahta et al. (2020) and Abd Razak et al. (2020). Briefly, a hyaluronidase solution was prepared at a concentration of 15 units/mL in 20 mM phosphate buffer (pH 7.0) containing 77 mM sodium chloride and 0.01% *w*/*v* of BSA. Subsequently, 100 μL of each cricket extract was incubated with 100 μL of hyaluronidase enzyme solution at 37 °C in the dark for 10 min. Following this, 100 μL of hyaluronic acid solution in 300 mM phosphate buffer (pH 5.35) was added, and the resulting mixture was further incubated at 37 °C for 45 min. After that, 1 mL of a 0.1 mg/mL acidic BSA solution in 24 mM acetate buffer (pH 3.75) was added, allowing the undigested hyaluronic acid to precipitate during incubation at ambient temperature for 10 min. The absorbance of the resulting mixture was measured at 600 nm using a microplate reader (CLARIOstar PLUS, BMG Labtech, Ortenberg, Germany). The hyaluronidase inhibition of each cricket extract was calculated using the following equation: Hyaluronidase inhibition (%) = (*A/B*) × 100, where *A* refers to the absorbance of the reaction consisting of extract, hyaluronidase, hyaluronic acid, and acidic BSA solution and *B* refers to the absorbance of the control reaction consisting of enzyme diluent, hyaluronic acid, and acidic BSA solution. Oleanolic acid served as a positive control. The IC_50_ values were calculated using GraphPad Prism (version 10.0, San Diego, CA, USA). All experiments were performed in triplicate.

### 2.6 Determination of the safety profile of cricket extracts by hen's egg test on chorioallantoic membrane (HET-CAM) assay

The irritating potential of each cricket extract was evaluated using the HET-CAM assay according to the method of Inthorn et al. (2024). Since fertile 7-day-old hen's eggs, which were in the early phase of embryonic development (days 3 to 7), were used in the current study, ethics committee approval was not required (Ozturk et al., 2020). In brief, 30 μL of the sample solution was applied to the CAM, and signs of irritation, including hemorrhage, vascular lysis, and coagulation, were assessed under a stereomicroscope (Olympus, Tokyo, Japan) for 5 min. The irritation score (IS) was calculated using the following equation: IS = [(301−*h*) × 5]/ 300 + [(301−*l*) × 7]/300 + [(301 - c) × 9]/300, where h, l, and c refer to the time in seconds of initial hemorrhage, vascular lysis, and coagulation, respectively. IS were categorized into four levels: non-irritation (0.0–0.9), mild irritation (1.0–4.9), moderate irritation (5.0–8.9), and severe irritation (9.0–21.0). Normal saline solution (0.9% *w*/*v* NaCl) served as a negative control, while SLS aqueous solution (1% *w*/*v*) served as a positive control. All experiments were performed in duplicate.

### 2.7 Development of nanoemulsions with and without TMS

#### 2.7.1 Preparation of nanoemulsions

The nanoemulsion was prepared using high-pressure homogenization, based on the method described by Chaiyana et al. (2024), with 5% *w*/w *T. mitratus* oil as the oil phase, 5% w/w Tween® 85 as the emulsifier, and 5% w/w sorbitol as the humectant (Chaiyana et al., 2024). In brief, all components, along with water as the continuous phase of the nanoemulsion, were mixed using a high-shear homogenizer (T25 ULTRA-TURRAX® digital, IKA Werke GmbH & Co. KG, Staufen, Germany) set at 12,000 rpm for 5 min at room temperature. Subsequently, the mixture underwent five cycles of high-pressure homogenization (APV 1000, Wilmington, MA, USA) at 300 bars. The TMS was selected based on its potency for cosmeceutical effects and safety profile. The concentration of the TMS was set at 0.1% w/w, selected based on its biological activity profile, corresponding to a level above its IC_50_ values to ensure sufficient biological activity. The TMS was dispersed in the *T. mitratus* oil and incorporated following the preparation method described above.

#### 2.7.2 Physicochemical characterization of nanoemulsions

Nanoemulsions with and without TMS were evaluated for their external appearance, droplet size, polydispersity index (PDI), zeta potentials, and morphology under a transmission electron microscope (TEM). The external appearance was evaluated through organoleptic inspection. Internal droplet size and PDI were assessed using dynamic light scattering with a Zetasizer (version 5.00, Malvern Instruments Ltd., Malvern, UK). Additionally, the zeta potential was analyzed using electrophoretic light scattering with the same instrument. Prior to all measurements, each nanoemulsion was diluted 10-fold with DI water. Data are presented as the mean ± standard deviation (SD), derived from 10 repeated measurements per run, with three independent runs per sample. The morphological characteristics of the nanoemulsion were analyzed using TEM. Prior to the TEM analysis, the nanoemulsion was diluted tenfold with DI water. A small volume of each diluted sample was then dropped onto a 300-mesh carbon-coated copper TEM grid. To enhance contrast, 1% *w*/*v* phosphotungstic acid was applied for negative staining. The grids were subsequently vacuum-dried for 30 min. TEM imaging was carried out at an accelerating voltage of 100 kV and a magnification of 20,000× using a JEOL JEM-2010 (JEOL Ltd., Tokyo, Japan) (Jandang et al., 2024).

#### 2.7.3 Stability of nanoemulsions

The physical stability of the nanoemulsions was assessed using the centrifugation technique (Chaiyana et al., 2024). The formulations were centrifuged using a microcentrifuge (MINI-10 K+, Hangzhou Miu Instruments Co., Ltd., Hangzhou, China) set at 5000 rpm for 15 min. After that, the external appearance, especially regarding phase separation, was assessed by visual inspection. Furthermore, all nanoemulsions that remained stable after centrifugation were further subjected to accelerated stability testing. This involved repeated temperature cycling, consisting of heating at 45 °C for 48 h followed by cooling at 4 °C for 48 h, for a total of six cycles. After completion of the cycles, the external appearance and any phase separation were evaluated by visual inspection. Droplet size, PDI, and zeta potential were evaluated following the method described in section 2.7.2.

### 2.8 Determination of entrapment efficiency (EE) and loading capacity (LC)

The nanoemulsion containing TMS was evaluated for the EE and LC using indirect methods (Silva et al., 2022; Yeerong et al., 2024a). In brief, an aliquot of the nanoemulsion was subjected to centrifugation using a centrifugal filter unit (Amicon Ultra-15, MWCO 100 kDa, Merck KGaA, Darmstadt, Germany) and a centrifuge (MPW-352R, MPW Med. instruments, Warsaw, Poland) set at 10,000 rpm for 15 min. During this process, the free (unentrapped) TMS passed through the filter membrane, while the entrapped portion remained in the retentate compartment. Therefore, the entrapped portion refers to protein that is not freely dissolved in the continuous aqueous phase but is instead associated with the nanoemulsion droplets, including proteins localized within the dispersed phase or adsorbed at the oil–water interface. Free protein remaining in the filtrate was considered non-entrapped. The filtrate was then collected and analyzed to quantify the free protein content using the BCA assay as described in section 2.4.1. EE and LC were calculated by subtracting the amount of free protein from the total amount initially added to the formulation following the equation: EE (% *w*/w) = [(*W*_*1*_ −*W*_*2*_)/*W*_*1*_] × 100 and LC (% w/w) = [(*W*_*1*_ −*W*_*2*_)/*W*_*3*_] × 100, where *W*_*1*_ represents the total protein content of the TMS incorporated into the nanoemulsion, *W*_*2*_ represents the total protein content of the filtrate, and *W*_*3*_ represents the mass of cricket oil. All experiments were performed in triplicate.

### 2.9 Release of TMS from the nanoemulsion

The release profiles of TMS from the nanoemulsion were evaluated in comparison with the aqueous solution using a dialysis bag technique (Gomez-Lazaro et al., 2024; Zhang et al., 2023). Prior to the experiment, dialysis bags with a molecular weight cut-off of 6–8 kDa (Spectra/Por®, Repligen Corp., Rancho Dominguez, CA, USA) were cut into small pieces and soaked in phosphate-buffered saline (PBS) at pH 5.5 overnight. Subsequently, the soaked dialysis bags were gently blot-dried using tissue paper, and one end was secured with coated aluminum wire. Then, 2 mL of each formulation was placed into the prepared dialysis bag, and the other end was carefully sealed with coated aluminum wire to avoid air bubble formation inside the bag. To start the experiment, each filled dialysis bag was placed into a beaker containing 20 mL of PBS pH 5.5, maintained on a magnetic stirrer (IKA® C-MAG HS7, IKA Werke GmbH & Co. KG, Staufen, Germany) set at 105 rpm and 32 °C. The release study was initiated once the sample was placed into the release medium. At predetermined time intervals of 0.5, 1, 2, 4, 6, 8, 24, and 48 h, 1 mL of the release medium was withdrawn and immediately replaced with an equal volume of fresh PBS pH 5.5 to maintain sink conditions. The released amount of the TMS was determined using a BCA assay as described in section 2.4.1. The receptor medium (PBS pH 5.5) was used as the blank to correct for background absorbance, and its value was subtracted from each sample measurement to obtain corrected protein concentrations. All experiments were performed in triplicate.

### 2.10 Determination of in vitro permeation and retention of TMS from the nanoemulsion

The permeation profiles and skin retention of TMS from the nanoemulsion were evaluated in comparison with an aqueous solution using vertical-type Franz diffusion cells (Velp Scientific Inc., Milano, Italy) (Arce et al., 2020; Ossowicz-Rupniewska et al., 2021). The Strat-M® membrane (Merck Millipore Ltd., County Cork, Ireland), a synthetic and non-animal-based membrane available as 25 mm discs, was selected as the skin model due to the absence of protein components, which minimizes interference with the analysis. In brief, the Strat-M® membrane was mounted between the donor and receptor compartments of the Franz diffusion cell. The receptor compartments were filled with PBS pH 7.4 and maintained at 32 °C. During the study, the receptor medium was continuously stirred at 400 rpm using a magnetic stirrer to maintain sink conditions. Subsequently, 1 g of the formulation was applied to the membrane in the donor compartment, which was selected to ensure complete and uniform coverage of the membrane surface and to maintain consistent donor phase conditions throughout the experiment without causing sample instability or uneven distribution. Aliquots of the receptor medium were withdrawn at predetermined intervals of 0.5, 1, 2, 4, 6, 8, and 24 h, with an equal volume of fresh PBS pH 7.4 replenished after each sampling. The amount of TMS in the receptor medium was quantified using the BCA assay as described in section 2.4.1. Following the 24-h permeation study, the remaining content of TMS in the Strat-M® membranes was evaluated. Each membrane was removed from the Franz diffusion cells and washed with DI water, cut into small pieces, and sonicated with PBS pH 7.4. The resulting extracts were centrifuged at 3200 ×*g* for 20 min, and the supernatants were collected for protein content determination using the BCA assay as described in section 2.4.1. All experiments were performed in triplicate. Further development of a cosmetic product incorporating a TMS-loaded nanoemulsion (Table S5), along with its preliminary single-arm (before–after) clinical evaluation of safety and efficacy, is presented in the Supplementary Data.

### 2.11 Statistical analysis

Data are presented as mean values with standard deviations. Statistical analysis was performed using GraphPad Prism (version 10.2.3, GraphPad Software Inc., La Jolla, CA, USA)., applying one-way ANOVA followed by Tukey's post hoc tests. The *p*-value of less than 0.05 was considered statistically significant.

## 3 Results and discussions

### 3.1 Potential of T. mitratus crude aqueous extract in skin-related applications

To evaluate the cosmeceutical potential of edible crickets, the amino acid composition, protein content, bioactivity, and safety of crude aqueous extracts from *G. bimaculatus*, *T. mitratu*s, and *A. domesticus* were compared, highlighting species-specific differences and the promising properties of *T. mitratu*s. The amino acid profiles of each cricket extract, as shown in Table S1, revealed total amino acid contents ranging from 67.9% to 77.0% of dry matter, with non-essential amino acids predominating. Glutamic acid, alanine, and aspartic acid were the most abundant non-essential amino acids, while leucine was the most prevalent essential amino acid. Crude aqueous extracts of the three cricket species exhibited comparable yields (13–14% *w*/w), as shown in Table S2, with *A. domesticus* showing the highest protein content. In vitro evaluation demonstrated that the *T. mitratus* extract exhibited the strongest anti-tyrosinase activity against both L-tyrosine (IC_50_ = 14.3 ± 10.5 μg/mL) and L-DOPA substrates (IC_50_ = 196.7 ± 15.5 μg/mL) and moderate anti-hyaluronidase activity (IC_50_ = 78.8 ± 8.5 μg/mL), indicating potential skin-brightening and anti-aging properties (Fig. S1 and Table S3). Safety assessment using the HET-CAM assay revealed that all crude extracts were non-irritating (Fig. S2 and Table S4). Therefore, these findings highlight *T. mitratus* as a promising candidate for further evaluations.

### 3.2 Enzyme-assisted extracts and protein isolates of T. mitratus

As *T. mitratus* crude aqueous extract demonstrated the most promising bioactivity in both anti-tyrosinase and anti-hyaluronidase assays, along with favorable safety profiles compared to other cricket extracts, further enzyme-assisted extraction and protein isolation from *T. mitratus* were undertaken to enhance these biological activities. Two methods of protein content determination were used for each cricket extract, including a BCA assay for comparative quantification, with results expressed as BSA equivalents, and a nitrogen analyzer for absolute measurement based on protein-to‑nitrogen conversion. Although BCA responses may vary between BSA and cricket-derived proteins, relative comparisons remain reliable under consistent conditions, and confirmation by the nitrogen analyzer further strengthens the accuracy of the protein determination. Yields and protein content of the cricket extracts are presented in Table 1. Among the extraction methods, enzyme-assisted extraction yielded the highest extract content, followed by crude aqueous extraction and protein isolates obtained through precipitation, respectively. Among various proteases enzymes, Subtilisin A produced the highest yield (59.3 ± 1.3% w/w, dry basis), which was significantly greater than the yields obtained using papain and trypsin (*p* < 0.05). The findings align with previous research demonstrating the superior hydrolytic efficiency of Subtilisin A, commercially known as Alcalase® (Cunha et al., 2025). As a serine endopeptidase, Subtilisin A cleaved peptide bonds within the central region of amino acid chains, and its broad substrate specificity combined with strong catalytic activity has consistently resulted in a high degree of protein hydrolysis across various protein sources (Tacias-Pascacio et al., 2020). Ahmadifard et al. (2016) reported that Subtilisin A exhibited approximately 10 times higher hydrolytic efficiency compared to papain and a commercial enzyme cocktail (trypsin, chymotrypsin, and aminopeptidase) during the enzymatic hydrolysis of rice bran and soybean protein concentrates. These observations support the current findings, where Subtilisin A also yielded the highest extraction efficiency among the enzymes tested. The increased yield observed with Subtilisin A could be attributed to its broader proteolytic activity, effectively breaking down the cricket biomass and releasing more extractable material.

In contrast to the extraction yields, the TMI exhibited the highest protein contents, as determined by both the BCA assay (91.9 ± 2.1% *w*/w) and nitrogen analysis (78.4 ± 0.1% w/w), indicating effective protein enrichment through the isolation process. Differences in protein content values obtained from the BCA assay and nitrogen analysis were observed, likely due to the varying sensitivities and principles underlying each method. However, it should be noted that protein concentrations determined by the BCA assay are expressed as BSA equivalents, as assay responses may vary between BSA and cricket-derived proteins. Nevertheless, since the comparisons in this study are primarily made between samples analyzed under the same conditions, the relative differences remain reliable. The BCA method, widely used for its simplicity and compatibility with microplate formats, relies on the reducing power of proteins in an alkaline environment (Walker, 2009). However, the BCA assay varies in sensitivity and accuracy depending on the sample matrix (Chang and Zhang, 2017; Sapan et al., 1999). The high purity of proteins in the isolates minimized matrix interference, resulting in the highest measured protein content. On the other hand, the nitrogen analyzer may underestimate protein content when other nitrogenous compounds are not fully decomposed during the combustion process (Cenci et al., 2021). After the protein isolates, the enzyme-assisted extract showed the highest protein content, whereas the crude aqueous extract had the lowest. Among the enzyme-assisted extracts, all protease enzymes yielded comparable protein contents as measured by the BCA assay, ranging from 76.0 ± 1.1% w/w to 76.1 ± 0.7% w/w. In contrast, nitrogen analysis indicated the highest protein content in the TMS (74.4 ± 0.3% w/w), followed by TMT (72.8 ± 0.0% w/w) and TMP (71.0 ± 0.7% w/w). The results were consistent with our results for the protein isolates, where the BCA assay showed higher protein contents, although the values were nearly identical. Consistent with previous reports, slight variations in measured protein content were observed depending on the assay used (Brooks et al., 1995). Differences in protein contents were detected by nitrogen analysis, indicating this method provides greater differentiation among samples. The likely explanation is that, based on the Dumas combustion method, the nitrogen analyzer measures total nitrogen and is widely regarded as a reliable method for protein quantification across various matrices (Jia et al., 2024). In contrast, the crude aqueous extract of *T. mitratus* yielded the lowest protein content among all extracts, with values from the BCA assay being lower than those obtained by nitrogen analysis. The likely explanation could be that the BCA assay, which relied on the reducing power of proteins in an alkaline environment, exhibited variability in sensitivity and accuracy depending on the sample matrix (Chang and Zhang, 2017; Sapan et al., 1999). Therefore, the complex composition of the crude aqueous cricket extract interfered with the assay, potentially leading to lower protein content measurements.

Among all *T. mitratus* extracts, the enzyme-assisted extracts and TMI contained significantly higher protein content compared to the crude aqueous extract (*p* < 0.05). This confirms that protease enzymatic treatment effectively enhanced protein extraction efficiency. Subtilisin A significantly improved both the extract yield and the protein content from *T. mitratus* (*p* < 0.05). Additionally, the markedly highest protein content in the TMI further demonstrates the effectiveness of the isolation procedure in concentrating protein fractions from the cricket material. However, the relatively low yield obtained from the protein isolation process may present a limitation for large-scale or industrial applications, where both efficiency and cost-effectiveness are critical. In contrast, enzyme-assisted extraction appears to be a more viable alternative, as it provides a more balanced outcome, achieving both a reasonable extraction yield and a relatively high protein content. This suggests that enzyme-assisted methods, particularly those involving Subtilisin A, could be better suited for applications that require efficient recovery of functional proteins from *T. mitratus*.

### 3.3 Cosmeceutical effects of enzyme-assisted extracts and protein isolates of T. mitratus

**Fig. 1:**
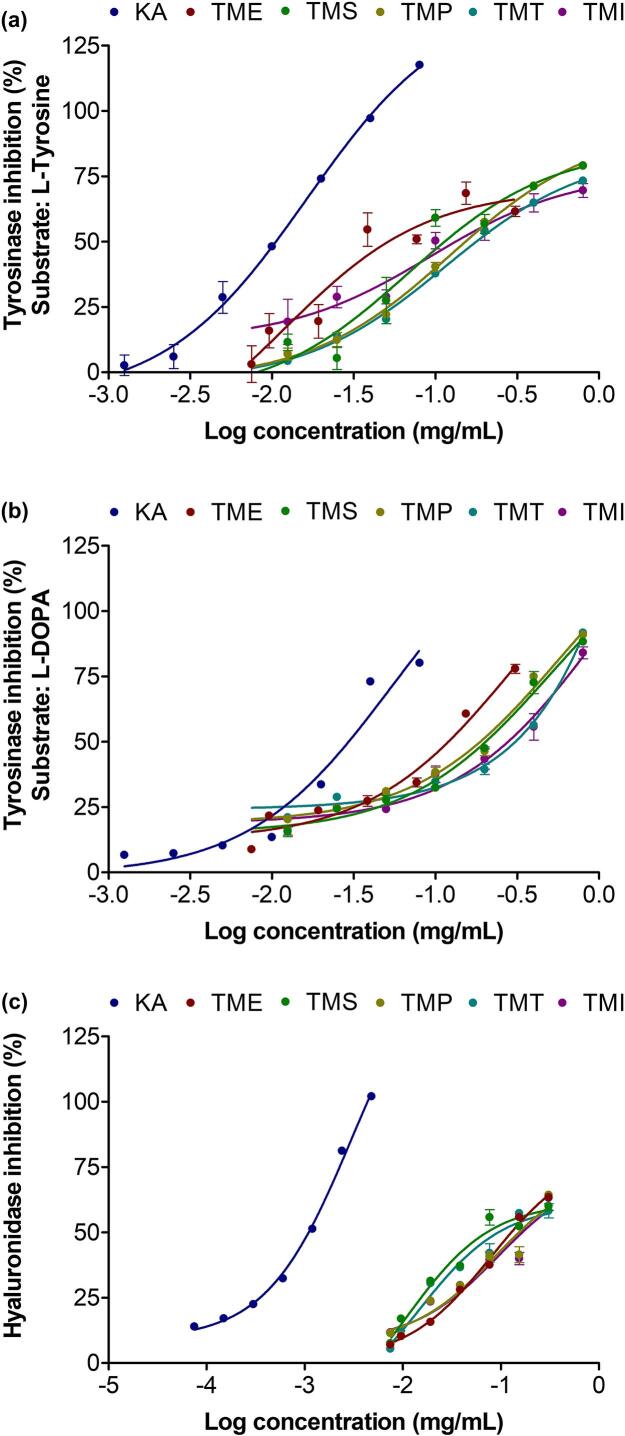
Dose–response curves represent the tyrosinase inhibition using L-tyrosine (a) and L-DOPA (b) as substrates, as well as the hyaluronidase inhibition (c) of kojic acid (KA), oleanolic acid (OA), and crude aqueous extracts from *T. mitratus* (TME), along with its enzyme-assisted extract using subtilisin A (TMS), papain (TMP), trypsin (TMT), and protein isolates (TMI). Data are presented as mean ± SD from three independent experiments, *n* = 3.

The cosmeceutical potential of various *T. mitratus* extracts was evaluated based on their inhibitory activities against tyrosinase and hyaluronidase enzymes. The dose-response curves of various *T. mitratus* extracts against tyrosinase activity using both substrates are shown in Fig. 1, while the corresponding IC₅₀ values are summarized in Table 2. All extracts, along with kojic acid as the positive control, exhibited dose-dependent inhibition of tyrosinase activity. IC₅₀ values were used to compare the potency of each sample, with lower IC₅₀ values indicating stronger inhibitory activity. Compared to the crude aqueous extract, the enzyme-assisted extracts and protein isolates demonstrated significantly reduced tyrosinase inhibitory activity. The observed reduction in tyrosinase inhibitory activity in enzyme-assisted extracts and protein isolates, relative to the crude aqueous extract, could be attributed to the partial degradation or modification of active compounds during processing. Tyrosinase-inhibitory peptides typically contain arginine and/or phenylalanine, often combined with hydrophobic residues like valine, alanine, or leucine (Schurink et al., 2007). In contrast, peptides rich in aspartic or glutamic acid tend to show poor binding, while lysine is not strongly associated with inhibition (Schurink et al., 2007)*.* Therefore, protein hydrolysis may alter the structural integrity of certain bioactive compounds, potentially leading to a loss of inhibitory function or reduced accessibility to the tyrosinase active site. This is particularly relevant for the amino acid profile of *T. mitratus* (Table S1), which includes amino acids associated with strong tyrosinase inhibition as well as those linked to weaker inhibitory effects. The superior performance of the Subtilisin A-treated extract suggested that this enzyme effectively generated or preserved peptides rich in tyrosinase-inhibitory amino acids, highlighting its potential for producing bioactive compounds for cosmetic use. Therefore, although some loss of activity may occur during enzymatic treatment, the careful selection of protease, such as Subtilisin A, can help retain or even enhance the generation of functionally relevant peptides.

In contrast, when considering hyaluronidase inhibition, enzymatic treatment appeared to enhance activity, as all enzyme-assisted extracts exhibited lower IC₅₀ values than the crude aqueous extract and TMI. This suggests that enzymatic hydrolysis contributed to the release of specific bioactive components with anti-hyaluronidase properties. The findings are consistent with the previous study by Yeerong et al. (2024c), which reported that *A. domesticus* protein hydrolysate exhibited the strongest anti-hyaluronidase activity, followed by the protein concentrate obtained via isoelectric precipitation and the crude aqueous extract (Yeerong et al., 2024c). The current study observed a similar trend in a different cricket species, *T. mitratus*, further supporting the potential of cricket-derived protein hydrolysates as effective hyaluronidase inhibitors. The hydrolysate likely inhibited hyaluronidase through competitive binding, preventing the enzyme from accessing its natural substrate (Chen et al., 2020). This suggested that specific peptides generated during hydrolysis may have structural affinity for the enzyme's active site. However, it should be noted that specific peptides were not identified in the current study, so these mechanistic interpretations remain hypothetical. Such interactions could potentially contribute to the activity of protein hydrolysates, highlighting their possible role as functional ingredients in cosmeceutical applications targeting skin aging.

### 3.4 Safety profile of enzyme-assisted extracts and protein isolates of T. mitratus

**Fig. 2:**
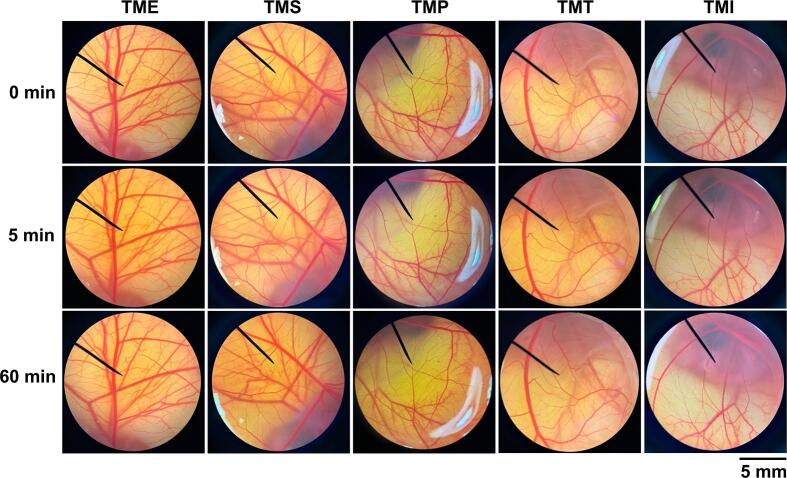
Effect of crude aqueous extract from *T. mitratus* (TME), enzyme-assisted extracts using subtilisin A (TMS), papain (TMP), and trypsin (TMT), and protein isolate (TMI) on the chorioallantoic membrane after 0, 5, and 60 min of exposure.

The irritation potential of *T. mitratus* extracts was evaluated using the HET-CAM assay, with the corresponding CAM images presented in Fig. 2. None of the tested extracts caused any visible irritation within 5 min or after 60 min of exposure, resulting in an IS of 0.0 ± 0.0. These findings confirm the non-irritating nature of all *T. mitratus* extracts.

### 3.5 Nanoemulsions with and without TMS

The TMS was selected due to its superior performance across multiple evaluation criteria, including the highest yield (Table 1), high protein content (Table 1), remarkable tyrosinase inhibition (Table 2), along with the most potent anti-hyaluronidase activity (Table 2). The findings suggest promising whitening and anti-aging effects, with the combined properties making the TMS the most favorable candidate for further cosmetic applications. The highest IC_50_ value was 395.9 ± 77.2 μg/mL, equivalent to approximately 0.04% *w*/w. Therefore, a concentration of 0.1% w/w of the TMS was incorporated into the nanoemulsion, which was approximately 2.5 times higher than the highest IC_50_ value. This concentration was selected as an exploratory approach to ensure sufficient bioactive levels for detecting potential effects within the formulation.

**Fig. 3:**
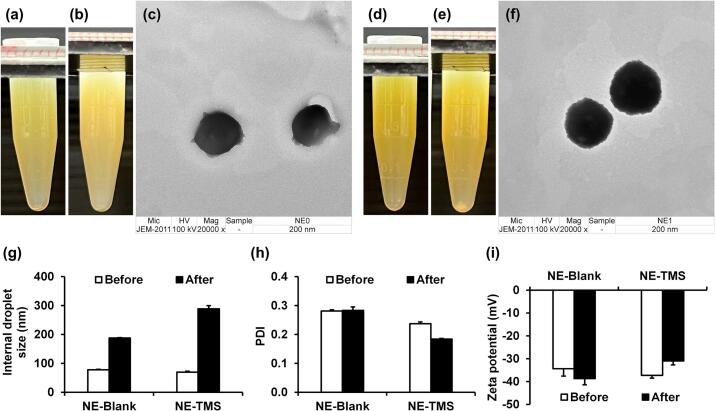
Visual appearance of the blank nanoemulsion before (a) and after centrifugation (b), and its morphology observed under TEM at a magnification of 6000× (c); visual appearance of the nanoemulsion containing 0.1% *w*/w TMS before (d) and after centrifugation (e), and its morphology under TEM at a magnification of 5000× (f). droplet size (g), polydispersity index (h), and zeta potential (i) of both nanoemulsions before and after the stability test under heating–cooling conditions. Data are presented as mean ± SD from three independent experiments, n = 3. Asterisks (*) indicate statistically significant differences at *p* < 0.05.

The TMS was incorporated into a nanoemulsion containing 5% w/w *T. mitratus* oil as the oil phase, following the nanoemulsion base previously developed by Chaiyana et al. (2024). The visual appearance of the blank nanoemulsion and the nanoemulsion containing TMS was homogeneous and translucent, with the latter showing a more intense yellow color, as shown in Fig. 3a and d, respectively. After centrifugation, no visible phase separation or sedimentation was observed in both nanoemulsion (Fig. 3b and e), confirming good physical stability. TEM further revealed spherical droplets with smooth surfaces and nanoscale dimensions for both formulations (Fig. 3c and f), supporting successful nanoemulsion formation. Nonetheless, it is important to note that TEM analysis without cryo-preservation may introduce some artifacts in hydrated systems, and the observed droplet size and morphology may be slightly influenced by deformation during sample preparation and vacuum conditions. Therefore, these observations are best interpreted as supportive and complementary to other characterization techniques. However, the droplet size of both formulations increased significantly after the heating–cooling stability test (Fig. 3g). The blank formulation increased from 78 ± 2 nm to 188 ± 3 nm, while the nanoemulsion containing TMS increased from 70 ± 1 nm to 288 ± 11 nm (*p* < 0.05). This enlargement likely resulted from droplet coalescence or structural rearrangements under thermal stress, with extract-loaded systems showing greater sensitivity. Although both formulations exhibited a significant increase in droplet size after the heating–cooling stability test, the final droplet sizes remained within the acceptable nanometer range for dermal delivery, as colloids sized between 50 and 500 nm are typically considered suitable (Uchechi et al., 2014). This indicated that the systems retained their nano-characteristics and physical integrity despite thermal stress. However, the greater size enlargement observed in the extract-loaded system suggested that the presence of *T. mitratus* protein may influence interfacial stability, possibly through interactions with surfactants or the oil phase. While the formulations retained their nano-characteristics over the short term, the observed droplet size increase during thermal cycling indicates moderate sensitivity to stress. Further evaluation under long-term storage conditions, including variations in temperature, humidity, and light exposure, would be necessary to confirm their robustness and shelf-life for practical applications.

On the other hands, their PDI values were below 0.3 (Fig. 3h), indicating narrow size distribution and high uniformity (Danaei et al., 2018). Importantly, PDI values remained stable after the heating–cooling test, suggesting that droplet growth did not lead to broad size variability or aggregation. Moreover, the zeta potential values of both nanoemulsions were higher negative values than −30 mV (Fig. 3i), indicating sufficient electrostatic repulsion to prevent droplet aggregation and ensure colloidal stability (Kumar and Mandal, 2018). No significant changes were detected after the stability test, further confirming the stability of the formulations. Therefore, these findings demonstrate that incorporation of TMS into nanoemulsions preserved physical stability, although droplet size increased after thermal cycling. The consistently low PDI and negative zeta potential values support their potential use as delivery systems for cosmeceutical applications. However, the observed sensitivity of droplet size to thermal stress suggested that careful attention to storage and handling conditions is required.

### 3.6 Entrapment efficiency and loading capacity of TMS in the nanoemulsion

EE and LC of TMS-loaded nanoemulsions are presented in Table 3. The formulation exhibited an EE of 46 ± 2% *w*/w, indicating that nearly half of the added TMS was successfully incorporated into the nanoemulsion system, possibly at the oil–water interface (Ji et al., 2015). The hydrophilic nature of the active protein versus the lipophilic internal oil phase can reduce EE and may compromise the bioactivity of the encapsulated protein (Massignan et al., 2024). Previous research also noted that insect-derived proteins can achieve moderate encapsulation, as *A. domesticus* protein hydrolysate loaded into chitosan–alginate nanoparticles for dermal delivery reached an EE of 56 ± 2% (Yeerong et al., 2024a). On the other hand, the LC of the formulation was determined to be 0.20 ± 0.01% w/w, reflecting the proportion of the extract relative to the total weight of the nanoemulsion droplets. This highlighted a known challenge of using nanoemulsions for hydrophilic protein extracts, as their solubility in the aqueous phase can limit loading capacity. Although the LC was low, it was consistent with typical nanoemulsion systems, where the internal oil phase and surfactant often limit the maximum payload of hydrophilic or bioactive extracts. However, even with a relatively low EE and LC, the nanoemulsion may still provide beneficial cosmetic effects, particularly if the active components are highly potent. Nanoemulsions could enhance skin contact, improve hydration, and allow gradual release, enabling the extract to exert its biological effects even at low concentrations (Iskandar et al., 2024).

### 3.7 Release of TMS from the nanoemulsion

**Fig. 4:**
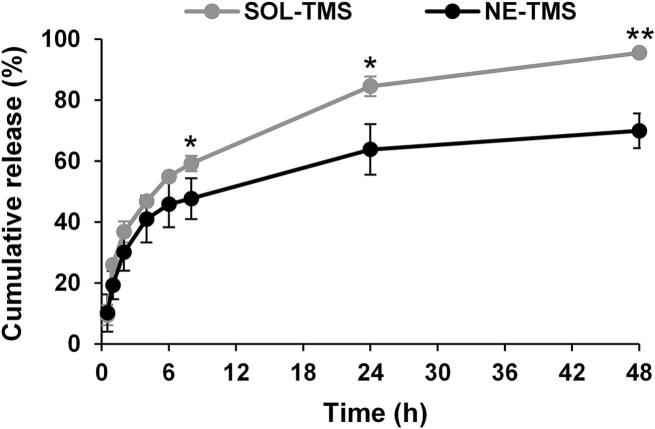
Release profile of TMS from solution (SOL-TMS) and nanoemulsion (NE-TMS). Asterisks denote significant difference between SOL-TMS and NE-TMS. Data are presented as mean ± SD from three independent experiments, n = 3 (* *p* < 0.05 and ** *p* < 0.01).

The cumulative release profiles of TMS from the nanoemulsion, in comparison with the aqueous extract, are shown in Fig. 4. The solution exhibited a rapid release, reaching approximately 55 ± 8% within 6 h and 95 ± 6% by 48 h. In contrast, the nanoemulsion showed a slower and more sustained release, achieving only 46 ± 1% at 6 h and 70 ± 1% at 48 h. Statistically significant differences between the two systems, confirming that the nanoemulsion effectively modulates release kinetics (*p* < 0.05 and *p* < 0.01). The slower release from the nanoemulsion can be attributed to the encapsulation of the extract within the nanoemulsion droplets, which acted as a barrier to immediate diffusion. This sustained release profile would be desirable for cosmeceutical applications, as it allows prolonged contact of the bioactive components with the skin, potentially enhancing efficacy and reducing the frequency of application (Casanova and Santos, 2016).

### 3.8 In vitro membrane permeation and retention of TMS from the nanoemulsion

The permeation and retention of TMS from nanoemulsion and aqueous solution were evaluated on an artificial Strat-M® membrane resembling skin using Franz diffusion cells. The results demonstrated that no detectable amount of the extract permeated into the receptor compartment of the Franz cells in either formulation, indicating that the extract may have limited ability to cross the barrier under these experimental conditions. This finding implies a low likelihood of systemic absorption, which may help minimize the risk of unwanted systemic side effects and support the potential safety for dermal use (Alikhan and Maibach, 2011). However, it should be noted that Strat-M® membranes are an in vitro model and cannot fully replicate human skin. Therefore, definitive conclusions regarding systemic absorption cannot be drawn without further studies using ex vivo skin models or clinical study.

**Fig. 5:**
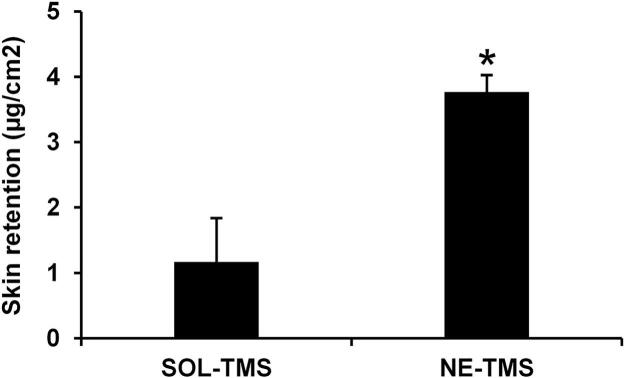
Strat-M® membrane retention of TMS from solution (SOL-TMS) and nanoemulsion (NE-TMS). Data are presented as mean ± SD from three independent experiments, n = 3. Asterisks denote significant difference between SOL-TMS and NE-TMS (* *p* < 0.05).

Although no permeation was observed, a notable difference was found in terms of membrane retention, as shown in Fig. 5. The nanoemulsion formulation demonstrated a significantly greater ability to retain TMS within the Strat-M® membrane compared to the aqueous extract solution, with retention values of 3.8 ± 0.3 μg/cm^2^ and 1.2 ± 0.7 μg/cm^2^, respectively (p < 0.05). These results highlight the potential of the nanoemulsion as an effective delivery system for improving the localization of TMS within the Strat-M® membrane model without significant transmembrane permeation. As Strat-M® is designed to mimic human skin permeability (Haq et al., 2018), these findings could provide preliminary evidence supporting the suitability of the nanoemulsion for cosmetic and dermatological applications that require high local activity but low systemic exposure. In contrast, the lower retention observed with the solution suggested a limited ability of the extract to interact with and remain within the Strat-M® membrane when delivered without a carrier system, likely due to the hydrophilic nature of the active proteins from *T. mitratus*. These results strongly highlighted the potential of nanoemulsion as an effective delivery system to enhance localization of TMS within the membrane model, which may be beneficial for cosmetic applications requiring localized delivery. However, it must be noted that although Strat-M® membranes resemble human skin and are widely used in place of actual skin, they do not fully replicate the biological complexity of human skin. Therefore, further evaluation would be needed to confirm these findings in actual human skin. The clinical evaluation of the cosmetic product containing the nanoemulsion of TMS (Fig. S3) was conducted as a preliminary, single-arm (before–after) study and is provided in the Supplementary Data (Section 5–6: Figs. S4 and S5).

## 4 Conclusions

The present study demonstrates that *T. mitratus* protein extracts possess significant cosmeceutical potential, combining skin-brightening and anti-aging effects. Among the extraction methods investigated, the TMS showed the highest bioactivity, with tyrosinase inhibition (IC_50_ = 65.4 ± 6.9 μg/mL for L-tyrosine and 395.9 ± 77.2 μg/mL for L-DOPA) and potent anti-hyaluronidase activity (IC_50_ = 12.5 ± 3.5 μg/mL). These results indicate effective suppression of melanogenesis and preservation of hyaluronic acid, which provides a mechanistic explanation for improvements in skin brightness, hydration, and elasticity observed in clinical studies. Incorporation of the TMS into an oil-in-water nanoemulsion enabled effective delivery and sustained release of the bioactive components. The nanoemulsion achieved an EE of 46.36 ± 1.69% and a LC of 0.20 ± 0.01% *w*/w, while maintaining nanoscale droplet size, uniform distribution (PDI < 0.3), and sufficient electrostatic stability (zeta potential < −30 mV). The nanoemulsion significantly increased retention of TMS (3.8 ± 0.3 μg/cm^2^) in the Strat-M® membrane, suggesting potential localization in upper layers of the skin avoiding systemic absorption. The study highlights the use of aqueous and enzyme-assisted *T. mitratus* extracts. The combination of multifunctional bioactivity, effective nanoemulsion delivery, and favorable safety profile indicates the potential of TMS as an innovative ingredient for multifunctional cosmeceutical products aimed at hydration, skin brightening, and anti-aging.

## CRediT authorship contribution statement

**Jirasit Inthorn:** Writing – review & editing, Writing – original draft, Visualization, Methodology, Investigation, Funding acquisition, Formal analysis. **Pratthana Chomchalao:** Methodology, Investigation, Formal analysis. **Saranya Juntrapirom:** Methodology, Investigation, Formal analysis. **Watchara Kanjanakawinkul:** Supervision, Resources, Methodology. **Andrea Heinz:** Writing – review & editing, Writing – original draft, Supervision, Resources, Methodology. **Anette Müllertz:** Writing – review & editing, Writing – original draft, Supervision, Resources, Methodology. **Thomas Rades:** Writing – review & editing, Writing – original draft, Supervision, Resources, Methodology, Conceptualization. **Wantida Chaiyana:** Writing – review & editing, Writing – original draft, Visualization, Supervision, Resources, Project administration, Methodology, Funding acquisition, Data curation, Conceptualization.

## Declaration of competing interest

The authors declare that they have no known competing financial interests or personal relationships that could have appeared to influence the work reported in the paper. The authors declare that the research was conducted in the absence of any commercial or financial relationships that could be constructed as a potential conflict of interest.

## Data Availability

Data will be made available on request.
