# Supplementary material for: Biological activities and safety assessment of Teleogryllus mitratus extracts for skin delivery via nanoemulsion-based systems
Source: Int J Pharm X. 2026 Apr 10;11:100542. doi: 10.1016/j.ijpx.2026.100542 (PMC13092756; doi:10.1016/j.ijpx.2026.100542)
Supplement: Supplementary file 1 — Supplementary material [file mmc1.pdf]

Supplementary data

Biological Activities and Safety Assessment of *Teleogryllus mitratus* Extracts for Skin Delivery via Nanoemulsion-Based Systems

Jirasit Inthorn [a], Pratthana Chomchalao [b], Saranya Juntrapirom [c], Watchara Kanjanakawinkul [c], Andrea Heinz [d], Anette Müllertz [e,f], Thomas Rades [f], Wantida Chaiyana [a,g,h,i,*]

[a] Department of Pharmaceutical Sciences, Faculty of Pharmacy, Chiang Mai University, Chiang Mai 50200, Thailand

[b] College of Medicine and Public Health, Ubon Ratchathani University, Ubon Ratchathani 34190, Thailand

[c] Chulabhorn Royal Pharmaceutical Manufacturing Facilities by Chulabhorn Royal Academy, Chon Buri 20180, Thailand

[d] Department of Pharmacy, LEO Foundation Center for Cutaneous Drug Delivery, University of Copenhagen, 2100, Copenhagen, Denmark

[e] Bioneer: FARMA, Department of Pharmacy, University of Copenhagen, Universitetsparken 4, Copenhagen, 2100, Denmark

[f] Department of Pharmacy, Faculty of Health and Medical Sciences, University of Copenhagen, Universi-tetsparken 2, 2100, Copenhagen, Denmark

[g] Center of Excellence in Pharmaceutical Nanotechnology, Faculty of Pharmacy, Chiang Mai University, Chiang Mai 50200, Thailand

[h] Research Center of Deep Technology in Beekeeping and Bee Products for Sustainable Development Goals (SMART BEE SDGs), Chiang Mai University, Chiang Mai 50200, Thailand

[i] Multidisciplinary and Interdisciplinary School, Chiang Mai University, Chiang Mai 50200, Thailand; wan-tida.chaiyana@cmu.ac.th (W.C.)

*Correspondence: wantida.chaiyana@cmu.ac.th; Tel.: +66-53944343

1. Amino acid profile of cricket dried materials

The amino acid profile of the defatted cricket powder was analyzed following the method and procedure outlined in the official methods of analysis of the Association of Official Analytical Collaboration International (1995) (Cunniff and Washington,1997). In brief, the defatted cricket powder underwent oxidation using performic acid and subsequent hydrolysis with 6 M HCl. The hydrolysates were diluted with sodium citrate buffer, neutralized, and the pH was adjusted to 2.20 before analysis. A separate procedure was used for tryptophan analysis, where the defatted cricket powder samples were hydrolyzed with 4.2 M NaOH under vacuum, followed by pH adjustment and clarification (Cevikkalp et al., 2016). The individual amino acids in cricket powder were detected using gas chromatography (GC model 6890 N, Agilent, Waldbronn, Germany) coupled with MS detection (model 5973 Inert, Agilent Technologies, CA, USA) and a Zebron ZB-AAA column (10 m × 0.25 mm, 0.25 μm film thickness, Phenomenex, Torrance, CA, USA).

The amino acid profiles of various cricket species are presented in Table S1. In the current study, the amino acid content of *G. bimaculatus*, *T. mitratus*, and *A. domesticus* ranged from 67.86% to 77.04%. These results are consistent with the findings of Udomsil et al. (2019), who reported that the crude protein content of crickets ranged from approximately 60% to 70% of dry weight. In some studies, crickets have been reported to be rich in protein, with a wider range of 55–73% conditions (Magara et al., 2020) or 55–69% (Kuo and Fisher, 2022). Variations in protein content among different cricket species can be attributed to factors such as species differences, sources, habitat, diet, developmental stage, and environmental conditions (Magara et al., 2020). The current study revealed that *A. domesticus* contained the highest amino acid content (77.04% w/w), followed by *T. mitratus* (72.6% w/w) and *G. bimaculatus* (67.86% w/w). The findings were consistent with those of Udomsil et al. (2019), who reported that *A. domesticus* contained a higher protein content (71.7 ± 0.5% w/w) than *G. bimaculatus* (60.7 ± 0.4% w/w). In contrast, Perera et al. (2025) reported differing findings, with *G. bimaculatus* exhibiting the highest protein content (58.0 ± 2.8% w/w), followed by *T. mitratus* (50.1 ± 1.3% w/w) and *A. domesticus* (48.4 ± 1.4% w/w). Perera and Bhujel (2022) also reported that *A. domesticus* had a lower protein content than *G. bimaculatus*, with levels of 48.4% w/w and 58.0% w/w of dry matter, respectively. On the other hand, some studies reported comparable protein contents between *A. domesticus* and *G. bimaculatus*, with the levels of 56.8% w/w and 53.4% w/w, respectively (Orkusz et al., 2024). Variations in protein content among different cricket species may be attributed to other factors than species, such as origin, habitat, diet, developmental stage, and environmental conditions (Magara et al., 2020).

Table S1. Amino acid profiles of different cricket species

Amino acids	Amino acid content (% w/w)		
	G. bimaculatus	*T. mitratus*	*A. domesticus*
Leucine	5.23	5.65	5.53
Valine	4.57	4.82	4.94
Lysine	2.77	3.06	3.74
Isoleucine	2.53	2.74	2.89
Phenylalanine	2.33	2.44	2.59
Threonine	2.34	2.41	2.81
Histidine	1.80	1.90	1.95
Methionine	1.05	1.18	1.23
**Essential amino acids**	**22.62**	**24.20**	**25.68**
Glutamic acid	8.06	9.10	8.76
Alanine	6.05	6.53	6.40
Aspartic acid	5.53	5.86	6.38
Arginine	3.73	4.04	4.26
Tyrosine	3.47	3.79	3.81
Glycine	3.45	3.71	3.62
Proline	2.99	3.23	3.87
Serine	2.68	2.58	3.30
Tryptophan	0.52	0.61	0.61
Cysteine	N.D.	N.D.	0.31
**Non-essential amino acids**	**45.24**	**48.40**	**51.36**
**Total amino acid content**	**67.86**	**72.6**	**77.04**

N.D. refers to an amount less than the limit of quantification of 0.20% of the defatted powder.

Among the amino acids, non-essential amino acids were found in higher amounts than essential amino acids, and the abundance ranking of both types was consistent with their respective contributions to the total amino acid content. Essential amino acids are those that cannot be synthesized de novo in sufficient quantities by the body and must be acquired through the diet to support maintenance, growth, and overall health (Hou et al., 2015). In contrast, non-essential amino acids can be synthesized by the body in adequate amounts and, therefore, do not need to be obtained from dietary sources (Hou et al., 2015). However, non-essential amino

acids play crucial roles that essential amino acids cannot fulfill, and their synthesis may not be sufficient to meet optimal metabolic and functional needs under both normal and stress conditions (Hou and Wu, 2017). Therefore, the higher content of non-essential amino acids is essential and aligns with typical patterns observed in animal-derived proteins, such as those found in pigs, cattle, sheep, and chickens (Hou and Wu, 2017). Among the non-essential amino acids, glutamic acid was identified as the most abundant across all investigated cricket species. This was followed by alanine and aspartic acid, which also ranked highly among the non-essential amino acids. These findings are consistent with previous studies, which have similarly reported glutamic acid as the predominant amino acid in various cricket species (Udomsil et al., 2019; Choi et al., 2017; Ghosh et al., 2017; Ganguly et al., 2021). Among the essential amino acids, leucine was present in the highest amount, following the three most abundant non-essential amino acids.

As crickets are revealed to be a good source of amino acids, they are attractive for skin applications, as amino acids are the building blocks of all proteins, including the most abundant fibrous proteins in the skin, such as keratin, collagen, and elastin (Solano, 2020). Additionally, amino acids, peptides, and proteins are well-known as classical moisturizers used to treat dryness in photoaged skin and reduce superficial skin folds (Mohiuddin, 2019). Glutamic acid has been reported to influence skin biology by decreasing the expression of apoptosis-related genes and enhancing cell viability and proliferation in human keratinocyte cultures (Jara et al., 2021). Glutamic acid has also been shown to act on damaged skin by accelerating barrier recovery, collectively suggesting a beneficial role in skin repair (Fuziwara et al., 2003). The high content of glutamic acid in the crickets suggests a promising potential to promote skin hydration, barrier repair, and regeneration when incorporated into cosmetic formulations. On the other hand, alanine has been found to stimulate the biosynthesis of nucleic acids and collagen (Nagai et al., 1986). Moreover, alanine, a key component of L-carnosine, has been reported to support muscle buffering capacity, prevent age-related muscle loss, and contribute to the prevention of skin aging (Gasmi et al., 2025). On the other hand, aspartic acid derivatives, such as acetyl aspartic acid, offer enhanced bioactivity and stability, promoting keratinocyte regeneration, inhibiting the expression of dermal MMPs, and reducing fibroblast stiffness, thereby improving skin elasticity and supporting overall skin health (Duracher et al., 2015; Gillbro et al., 2015). Leucine has been reported to promote fibroblast proliferation and protein synthesis, and its supplementation exerts an anabolic effect on protein metabolism, enhancing tissue repair and regeneration in skin wounds and muscles (Zhang et al., 2004).

2. Crude aqueous extracts and their protein contents

Each defatted cricket powder sample (30 g) was extracted in 300 mL of DI water under continuous agitation using a magnetic stirrer (IKA® C-MAG HS7, IKA Werke GmbH & Co. KG, Staufen, Germany) set at 500 rpm for 4 h at room temperature. The resulting mixture was centrifuged at 3,200 × g for 20 min using a MPW-352R, centrifuge (MPW MED. INSTRUMENTS, Warsaw, Poland). The supernatant was collected and freeze-dried using a CHRIST Beta 2-8 LDplus freeze dryer (Martin Christ Gefriertrocknungsanlagen GmbH, Osterode am Harz, Germany). The crude extracts from *G. bimaculatus*, *T. mitratus*, and *A. domesticus* were kept in a sealed aluminum foil bag until further experimentation.

The total protein content of each cricket extract was determined using a BCA assay, following the method described by Mba et al. (2021) and nitrogen analyzer according to the Dumas combustion method (Dumatherm N Pro, Gerhardt GmbH & Co. KG, Königswinter, Germany). Regarding the BCA assay, 25 µL of each sample solution was mixed with 200 µL of BCA working reagent and incubated at 37 °C for 30 min. The absorbance was then measured at 562 nm using a microplate reader (CLARIOstar PLUS, BMG Labtech, Ortenberg, Germany). The absorbance values of each sample were used to calculate the total protein content, expressed as g BSA/g extract, based on the equation of the standard curve plotted from absorbance values against BSA concentration.

Besides, the nitrogen content of each cricket extract was analyzed using a nitrogen analyzer according to the Dumas combustion method (Dumatherm N Pro, Gerhardt GmbH & Co. KG, Königswinter, Germany). In brief, 20 mg of each cricket extract was placed on the sample feeding plate and combusted in a reactor at 1030 °C using helium, nitrogen, and oxygen as carrier gases. EDTA was used as a standard substance to calibrate the nitrogen content (Choi et al., 2017; Quinteros et al., 2022). The resulting nitrogen weight was recorded and converted to the protein content using a standard protein-to-nitrogen conversion factor of 6.25 (Choi et al., 2017). The total protein content of each extract was calculated using the following equation: Total protein content (%) = $[(A/B) \times 100] \times 6.25$, where A refers to the weight of nitrogen and B refers to the weight of the cricket extract. All experiments were performed in triplicate.

The extraction yields of crude aqueous extracts from different cricket species are shown in Table S2. The yields from *G. bimaculatus*, *T. mitratus*, and *A. domesticus* were comparable, ranging from 13.1 ± 0.7 to 14.1 ± 0.3% w/w on a dry basis. In contrast, the protein content assessed using both the BCA assay and nitrogen analysis exhibited consistent trends, with *A. domesticus* showing significantly higher protein levels than the other two species ($p < 0.05$). The agreement between the BCA assay and nitrogen analysis confirmed the reliability of

protein quantification, although slight variations were noted. A likely explanation for the variations is that the BCA assay measures peptide bonds and aromatic amino acids, while nitrogen analysis quantifies total nitrogen content, which may include non-protein nitrogenous compounds (Lovrien and Matulis, 1995). Additionally, the findings regarding protein content were consistent with a previous report, which noted that *A. domesticus* and *G. bimaculatus* contained high amounts of protein, ranging from 60 – 70% on a dry weight basis, with a higher content observed in *A. domesticus* (71.7 ± 0.5%) compared to *G. bimaculatus* (60.7 ± 0.4%) (Udomsil et al., 2019).

Table S2. Yields and protein content of crude aqueous extracts

Yields and protein content	*G. bimaculatus*	*T. mitratus*	*A. domesticus*
Yield (%w/w based on dry basis)	13.1 ± 0.7	13.9 ± 0.5	14.1 ± 0.3
Protein content			
• BCA assay	70.2 ± 1.4 [b]	65.7 ± 3.0 [b]	78.7 ± 4.1 [a]
• Nitrogen analysis	69.3 ± 0.4 [b]	71.0 ± 0.2 [b]	77.0 ± 1.7 [a]

The letters a and b indicate statistically significant differences among the crude aqueous extracts from various cricket species (one-way ANOVA, Tukey's post hoc test, $p < 0.05$).

3. Cosmeceutical effects of crude aqueous cricket extracts

The cosmeceutical effects of crude aqueous cricket extracts were evaluated based on their tyrosinase and hyaluronidase inhibitory activities, as both enzymes play key roles in important skin-related processes. Tyrosinase is a key enzyme in the biosynthesis of melanin, the primary pigment responsible for skin coloration (Solano et al., 2014). Dysregulation or excessive activity of tyrosinase leads to melanin overproduction, resulting in hyperpigmentation disorders (Serre et al., 2018). Consequently, tyrosinase inhibitors have become pivotal bioactive agents in dermatological formulations serving as effective components in skin-whitening and anti-dark spot cosmetic products (Manap et al., 2021). Tyrosinase primarily catalyzes two types of reactions essential to melanogenesis, including the hydroxylation of monophenols (L-tyrosine) to *o*-diphenols (L-DOPA) and the subsequent oxidation of these *o*-diphenols to their corresponding *o*-quinones (dopaquinone) and ultimately melanin (Fernandez-Julia et al., 2021). Therefore, L-tyrosine is used as a substrate to determine monophenolase activity, whereas L-DOPA serves as the substrate to assess diphenolase activity. Evaluating both activities provides an understanding of inhibitory effects on different

stages of melanin synthesis, as certain compounds may selectively inhibit either the initial or later steps of the pathway. The dose-response curves illustrating the inhibitory effects of crude aqueous cricket extracts on tyrosinase activity with both substrates are presented in Figure S1, with the corresponding IC_{50} values listed in Table S3. All crude aqueous cricket extracts, together with kojic acid as the positive control for anti-tyrosinase activity, demonstrated dose-dependent inhibitory effects. Kojic acid exhibited the significantly lowest IC_{50} values for both L-tyrosine and L-DOPA substrates (1.0 ± 0.1 µg/mL and 1.7 ± 0.1 µg/mL, respectively), indicating potent inhibitory efficiency ($p < 0.05$). Among the crude aqueous cricket extracts, the extract from *T. mitratus* exhibited the strongest tyrosinase inhibition, for both L-tyrosine and L-DOPA substrates (IC_{50} = 14.3 ± 10.5 µg/mL for L-tyrosine and 196.7 ± 15.5 µg/mL for L-DOPA). The findings revealed that crude aqueous extract from *T. mitratus* possessed superior monophenolase and diphenolase inhibitory potential among the tested extracts, supporting its use in formulations aimed at anti-melanogenesis or skin whitening.

The findings support previous research regarding the anti-tyrosinase potential of cricket extracts. However, while earlier studies primarily highlighted the efficacy of non-polar or semi-polar solvent extracts, the present study is the first to report the anti-tyrosinase activity of aqueous extracts, offering a greener and more sustainable alternative. Previous studies have demonstrated anti-tyrosinase activity in the hexane extract of *A. domesticus* and the methanolic extract of *G. bimaculatus*, both obtained through organic solvent extraction (Masoongnoen et al., 2023). Fatty acids such as hexanoic acid and octanoic acid were suggested to be responsible for this activity (Masoongnoen et al., 2023). Additionally, *G. bimaculatus* oil obtained via cold-press extraction has been reported to exhibit promising anti-tyrosinase activity, with IC_{50} values of 172.2 ± 43.9 µg/mL for L-tyrosine hydroxylation and 837.6 ± 194.5 µg/mL for L-DOPA oxidation (Chaiyana et al., 2023). Compared to the previous study, the crude aqueous cricket extracts in the current study demonstrated greater anti-tyrosinase potency. The extract from *G. bimaculatus* exhibited lower IC_{50} values of 36.5 ± 4.7 µg/mL for L-tyrosine hydroxylation and 600.9 ± 59.4 µg/mL for L-DOPA oxidation, indicating stronger inhibitory activity than the oil previously reported. Although the potential in tyrosinase inhibition of the crude aqueous cricket extracts was not as potent as kojic acid, the benefits of their natural origin, lower toxicity, and greater sustainability should be noted. Kojic acid, a well-known antimelanogenic agent, also has notable drawbacks, including cytotoxicity, risk of sensitization, and instability during storage (Singh et al., 2016; Couteau and Coiffard, 2016). Therefore, research into novel anti-tyrosinase compounds is of considerable interest. The current study suggests that crude aqueous cricket extracts from *T. mitratus* show the strongest tyrosinase inhibition. Although it was not

as potent as kojic acid, the IC_{50} values for both L-tyrosine and L-DOPA substrates (14.3 ± 10.5 μg/mL, equivalent to 0.00143% w/w for L-tyrosine, and 196.7 ± 15.5 μg/mL, equivalent to 0.0197% w/w for L-DOPA) indicate potential for use in cosmetic formulations.

Aside from the skin-whitening effect mediated by anti-tyrosinase activity, the anti-skin aging potential of crude aqueous cricket extracts was also evaluated by assessing their inhibitory activity against hyaluronidase. Hyaluronidase is an enzyme that degrades hyaluronic acid, a key component of the skin's extracellular matrix responsible for maintaining moisture, volume, and elasticity (Papakonstantinou et al., 2012). As excess hyaluronidase activity contributes to skin aging, dehydration, and wrinkle formation, its inhibition is therefore considered a key target in the development of anti-aging and moisturizing cosmeceuticals (Mohiuddin, 2019). The dose-response curves illustrating the inhibitory effects of crude aqueous cricket extracts on hyaluronidase activity are presented in Figure S1, with the corresponding IC_{50} values summarized in Table S3**.** Oleanolic acid (positive control) demonstrated strong inhibitory effects (IC_{50} = 2.4 ± 0.7 μg/mL). Among the crude aqueous cricket extracts, those from *G. bimaculatus* and *T. mitratus* displayed moderate inhibition (IC_{50} = 79.2 ± 6.8 and 78.8 ± 8.5 μg/mL, respectively), whereas *A. domesticus* extract showed significantly lower activity (IC_{50} = 240.3 ± 2.6 μg/mL) ($p < 0.05$). Although comparative research on the anti-hyaluronidase activity of different cricket species is limited, the crude aqueous extract of *A. domesticus* was found to be less potent than its extracts obtained using organic solvents such as hexane and ethanol (Yeerong et al., 2024a). Furthermore, protein concentrates obtained via isoelectric precipitation and protein hydrolysates have been reported to exhibit superior activity (Yeerong et al., 2024b). Therefore, protein concentrates and hydrolysates represent promising alternatives due to their efficacy enhancement and potential for greener, more sustainable cosmetic applications. Additionally, cricket extracts from *G. bimaculatus* and *T. mitratus*, which exhibited lower IC_{50} values, may be of particular interest. Notably, there have been no reports on the anti-hyaluronidase activity of their protein concentrates and hydrolysates, highlighting an area for further investigation. However, *T. mitratus* emerged as the most promising extract, exhibiting strong bioactivity in both anti-tyrosinase and anti-hyaluronidase assays, indicating its potential for skin brightening and anti-aging applications. Therefore, it was selected for further investigation for use in cosmetic formulations.

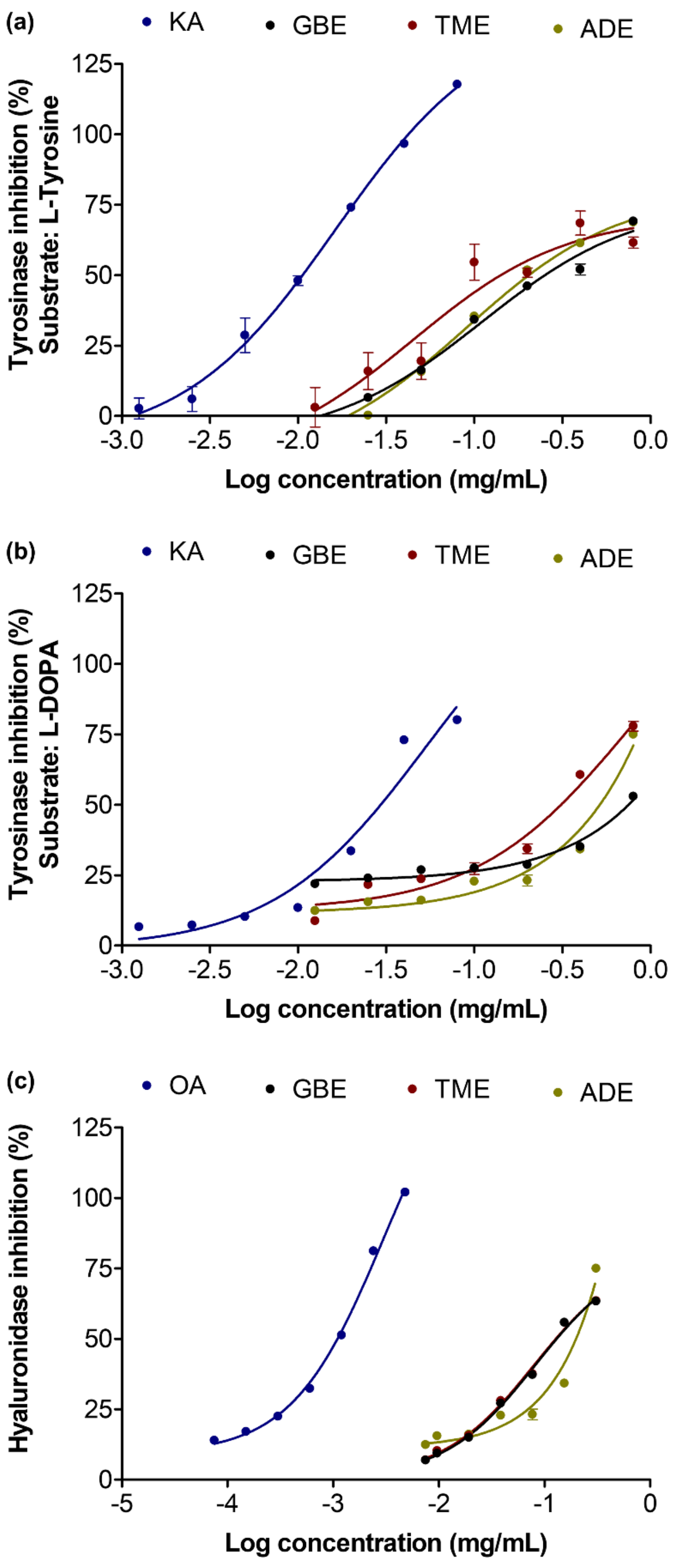

Figure S1. Dose–response curves representing tyrosinase inhibition using L-tyrosine (a) and L-DOPA (b) as substrates, as well as hyaluronidase inhibition (c) of kojic acid (KA), oleanolic acid (OA), and crude aqueous extracts from *G. bimaculatus* (GBE), *T. mitratus* (TME), and *A. domesticus* (ADE). Data are presented as mean ± SD from three experiments, n = 3.

Table S3. Cosmeceutical effects of crude aqueous cricket extracts and reference substances

Samples	Cosmeceutical effects: IC_{50} (µg/mL)		
	Anti-tyrosinase activity		Anti-hyaluronidase acctivity
	L-Tyrosine	L-DOPA	
Kojic acid	1.0 ± 0.1 [a]	1.7 ± 0.1 [a]	ND
Oleanolic acid	ND	ND	2.4 ± 0.7 [a]
GBE	36.5 ± 4.7 [c]	600.9 ± 59.4 [c]	79.2 ± 6.8 [b]
TME	14.3 ± 10.5 [a,b]	196.7 ± 15.5 [b]	78.8 ± 8.5 [b]
ADE	27.5 ± 5.1 [b,c]	346.1 ± 147.4 [b]	240.3 ± 2.6 [c]

GBE = crude aqueous extract of *G. bimaculatus*; TME = crude aqueous extract of *T. mitratus*, and ADE = crude aqueous extract of *A. domesticus*. ND refers to not determined. The letters a and b indicate statistically significant differences among the samples (one-way ANOVA, Tukey's post hoc test, $p < 0.05$).

3. Safety proflie of crude aqueous cricket extracts

The potential irritant effects of crude aqueous cricket extracts were assessed using the HET-CAM test, a widely accepted alternative to the Draize eye irritation test that has recently gained popularity for evaluating skin irritation potential (Steiling et al., 1999; Ma et al., 2021). The CAM exposed to the tested samples and their corresponding IS are presented in Figure S2 and Table S4, respectively. Exposure to a 1% w/v SLS aqueous solution, used as the positive control, resulted in hemorrhage, vascular lysis, and coagulation in the CAM. The calculated IS was 12.5 ± 0.3, indicating a severe level of irritation. Prolonged exposure to SLS further intensified these effects. In contrast, the negative control, normal saline (0.9% w/v NaCl), exhibited no signs of irritation, even after 60 min of exposure, with an IS of 0.0 ± 0.0.. When tested under the same conditions, all crude aqueous cricket extracts also showed IS values of 0.0 ± 0.0, suggesting no observable irritation. No visible changes were observed following 60 min of exposure to the extracts, indicating that all crude aqueous cricket extracts are non-irritating.

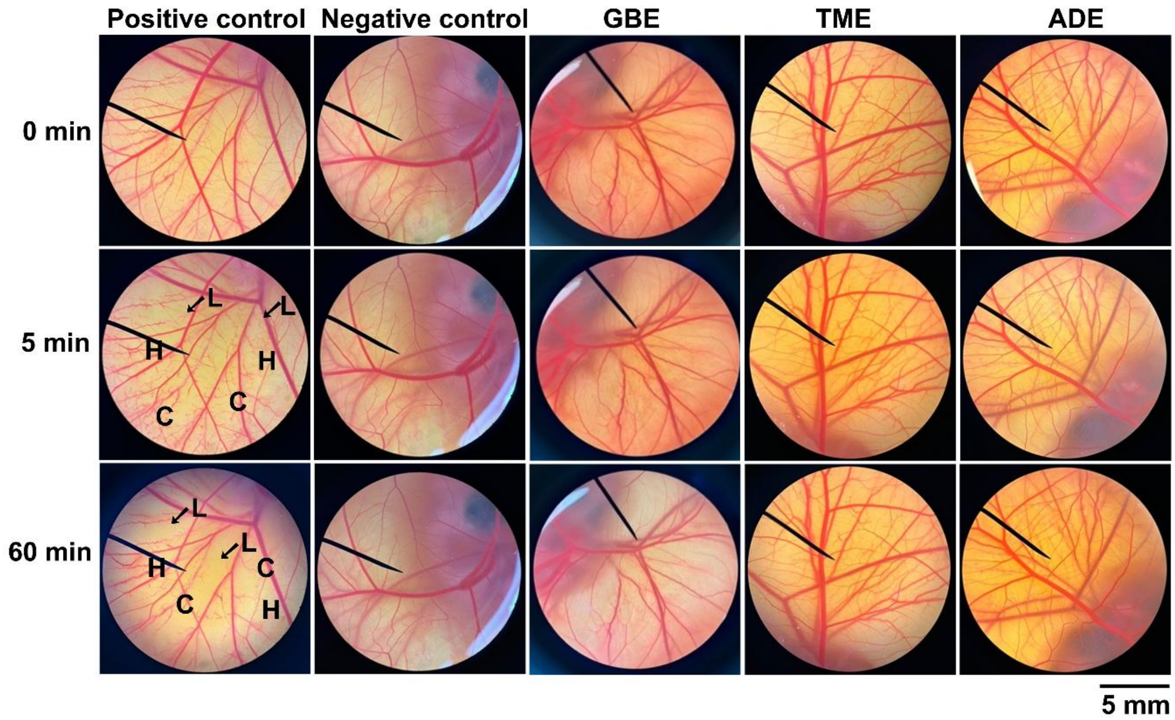

Figure S2. Effect of the positive control (1% w/v sodium lauryl sulfate aqueous solution), the negative control (normal saline solution), and the crude aqueous extracts obtained from *G. bimaculatus* (GBE), *T. mitratus* (TME), and *A. domesticus* (ADE) on the chorioallantoic membrane after 0, 5, and 60 min of exposure. H represents hemorrhage, L represents vascular lysis, and C represents coagulation.

Table S4. Irritation potential of crude aqueous extracts

Samples	Irritation Score	Irritation Potency
Positive control	19.6 ± 0.1 [a]	Severe irritation
Negative control	0.0 ± 0.0 [b]	No irritation
GBE	0.0 ± 0.0 [b]	No irritation
TME	0.0 ± 0.0 [b]	No irritation
ADE	0.0 ± 0.0 [b]	No irritation

GBE = crude aqueous extract of *G. bimaculatus*; TME = crude aqueous extract of *T. mitratus*, and ADE = crude aqueous extract of *A. domesticus*. Data are presented as mean ± SD from three independent experiments, n = 3. The letters a and b indicate statistically significant differences among the tested samples (one-way ANOVA, Tukey's post hoc test, $p < 0.05$).

4. Development of a cosmetic product containing nanoemulsion with TMS

A cosmetic product in the form of a facial serum containing 10% w/w nanoemulsion of TMS as the active ingredient was developed. The facial serum base was composed of various components as mentioned in Table S5. All ingredients were combined and mixed using a T25 ULTRA-TURRAX® digital homogenizer (IKA Werke GmbH & Co. KG, Staufen, Germany) set at 5,000 rpm for 15 min. Subsequently, the nanoemulsion of TMS was incorporated into the facial serum base formulation and mixed thoroughly a T25 ULTRA-TURRAX® digital homogenizer (IKA Werke GmbH & Co. KG, Staufen, Germany) set at 5,000 rpm for another 15 min.

Table S5. Composition of the cosmetic product formulation used in the clinical study

Ingredient (INCI name)	Function	Concentration (% w/w)
Rosa rubiginosa seed oil	Emollient	3.00
Hydrogenated polydecene	Emollient	1.00
Sodium polyacryloyldimethyl taurate	Emulsifier	0.60
Trideceth-10	Emulsifier	0.40
Glycerin	Humectant	3.00
Propanediol	Humectant	1.00
Ethylhexylglycerin	Preservative booster	1.00
Hyaluronic acid	Viscosity modifier	0.01
Xanthan gum	Viscosity modifier	0.10
Tocopheryl acetate	Antioxidant	1.00
Disodium EDTA	Chelating agent	0.05
Caprylhydroxamic acid	Preservative	0.15
1,2-Hexanediol	Solvent/Preservative	1.00
Butylene glycol	Solvent/Humectant	2.00
TMS nanoemulsion	Active ingredient	10.00
Purified water	Solvent	qs to 100

The cosmetic product containing nanoemulsion with TMS was characterized for its physicochemical properties, e.g., physical appearance, viscosity, and pH. The physical characteristics, including visual appearance and homogeneity, were evaluated through visual inspection. Additionally, the color of each formulation was evaluated using a HunterLab UltraScan VIS spectrophotometer (HunterLab, Inc., Reston, VA, US). Prior to the

measurement, an aliquot of the formulation was placed into an optically clear glass cell with a fixed path length of 10 mm (HunterLab, Inc., Reston, VA, US). The measurements were performed in the reflectance specular excluded mode. The results were recorded in terms of L*, a*, and b* values, where L* represents lightness (0 = black, 100 = white), a* indicates the red-green axis (positive values = red, negative values = green), and b* corresponds to the yellow-blue axis (positive values = yellow, negative values = blue) (Kowalska et al., 2021). The viscosity of each formulation was measured using a rheometer (MCR 102e, Anton Paar GmbH, Graz, Austria) equipped with a bob-and-cup geometry (bob length: 25 mm; gap: 2.25 mm) and a temperature control system (Julabo, CORIO CD-200 F, Seelbach, Germany) set at 23 ± 0.2 °C. The results expressed in centipoise (cP) was recorded at a fixed shear rate of 500 s^{-1} (Baptista et al., 2023). In addition, pH was measured using a pH meter (S220 SevenCompact, Mettler-Toledo International Inc., Greifensee, Switzerland). All measurements were performed in triplicate. The physical stability of the cosmetic product containing the nanoemulsion with TMS was evaluated using the centrifugation technique (Chaiyana et al., 2024) and under accelerated heating–cooling conditions for 60 days (Azeem et al., 2009). Visual inspection was performed after both the centrifugation and accelerated stability tests. Only the formulations that were stable after centrifugation and accelerated stability testing were further evaluated for color, viscosity, and pH.

A cosmetic product in the form of a facial serum was developed from the nanoemulsion with TMS. The visual appearance and physicochemical characteristics of the facial serum are presented in Figure S3. It should be noted that the gelification process involves mechanical re-processing, which may influence the structural integrity of the original nanoemulsion system. Therefore, the nanoemulsion structure in the final serum requires further experimental confirmation to verify the preservation of the nanoscale structure. The facial serum exhibited a uniform, milky-white, and homogenous appearance without any signs of phase separation or precipitation. The color parameters presented a high lightness value (L* = 75.24 ± 0.02) that reflected a bright and opaque formulation, an a* value close to zero suggesting no red or green tint, and a slightly positive b* value (10.58 ± 0.01) indicating a faint yellowish tone likely derived from the oil, emulsifier, and TMS. The viscosity of the serum was 244 ± 3 cP, representing a moderately viscous but easily spreadable consistency appropriate for topical application. Additionally, the pH was approximately 5.54 ± 0.03, which is close to the natural skin pH, making the formulation safe and well-suited for cosmetic use (Proksch, 2018).

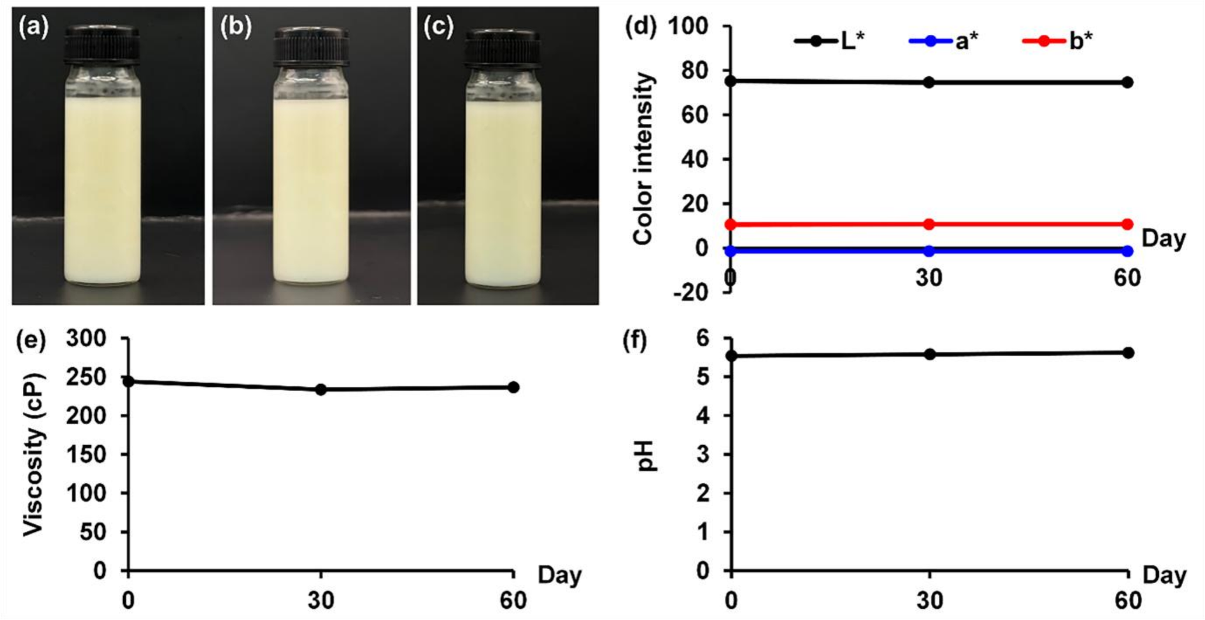

Figure S3. Visual appearance of facial serum containing nanoemulsion with TMS at day 0 (a), day 30 (b), and day 60 (c), along with their color parameters in terms of L*, a*, and b* (d), viscosity (e), and pH (f). Data are presented as mean ± SD from three experiments, n = 3.

The physical stability of the facial serum was evaluated over a storage period of 60 days. Visual observation revealed no significant phase separation or sedimentation throughout the study period, indicating that the formulation maintained its homogeneity and physical stability. Assessment of color stability using the parameters of L*, a*, b* showed that the values remained nearly constant throughout the 60-day storage period. This suggests that the nanoemulsion system effectively protected the active extract from degradation or oxidation that could otherwise cause noticeable color changes. Such stability is critical for consumer acceptance, as color variations in cosmetic products are often perceived as indicators of poor quality or instability (Romanowski and Schueller, 2001). The viscosity and pH of the formulation remained stable throughout the 60-day study period. These findings confirmed that the facial serum containing TMS-loaded nanoemulsion exhibited good physicochemical stability in terms of appearance, color, viscosity, and pH over 60 days.

5. Clinical evaluation of the cosmetic product containing nanoemulsion with TMS

The study protocols for clinical evaluation were reviewed and approved by the Human Research Ethics Committee of the Faculty of Pharmacy, Chiang Mai University, Thailand (Approval Certificate No. 009/2025/E). Thirty healthy Thai volunteers (23.3% male and 76.7% female) aged 20–60 years were enrolled according to predefined inclusion and exclusion

criteria. All clinical tests, including a treated site with a cosmetic product containing a nanoemulsion with TMS compared with an untreated control site (skin without treatment), were conducted on the forearm of the participants. Inclusion criteria comprised individuals with smooth forearm skin, free from any dermatological abnormalities such as hyperpigmentation, scars, or lesions. Exclusion criteria included a history of allergy to any component of the test product, ongoing medical treatments, pregnancy, or any skin conditions that could potentially affect product application or study outcomes. All participants were instructed to maintain their normal hygiene practices and usual use of cosmetic products during the study period.

The skin irritation potential of the cosmetic product containing nanoemulsion with TMS was assessed using the Finn chamber patch test, following the Organisation for Economic Co-operation and Development (OECD) guideline (2004) (Basketter et al., 2004). Briefly, 20 µL of the formulation was applied to 8 mm Finn chambers (SmartPractice Europe GmbH, Greven, Germany) mounted on Scanpor tape (SmartPractice Europe GmbH, Greven, Germany) and affixed to the outer upper forearm of each participant. The Finn chambers were detached after a 4-h exposure period, followed by assessments of skin reactions at 4, 24, 48, and 72 h after application. The primary dermal irritation index (PII) was calculated using the following equation: PII = $(A + B) / 4N$, where A denotes the cumulative erythema scores recorded at the four observation time points, B denotes the cumulative edema scores at the same time points, and N is the total number of subjects. PII values below 0.5 indicate non-irritation, values between 0.5 and 2.0 indicate mild irritation, 2.1 to 5.0 indicate moderate irritation, and values exceeding 5.0 signify severe irritation (Magrode et al., 2024). Aside from the cosmetic product containing nanoemulsion with TMS, 20% w/v SLS was used as a positive control, whereas DI water was used as a negative control. A facial serum formulated with a nanoemulsion containing TMS was subjected to clinical evaluation in 30 human volunteers. The safety of the facial serum was clinically evaluated. The results are shown in Figure S4, using 20% w/w sodium lauryl sulfate solution as the positive control and DI water as the negative control. No signs of erythema, itching, burning, or irritation were observed in the volunteers throughout the study period in both the negative control and the facial serum groups. The skin remained in a normal condition after the applications, confirming that the formulation was well tolerated. In contrast, the positive control induced signs of irritation, which were observed after 24 h and became more severe with longer exposure. Therefore, the facial serum was suggested to be safe for topical use and suitable for cosmetic application.

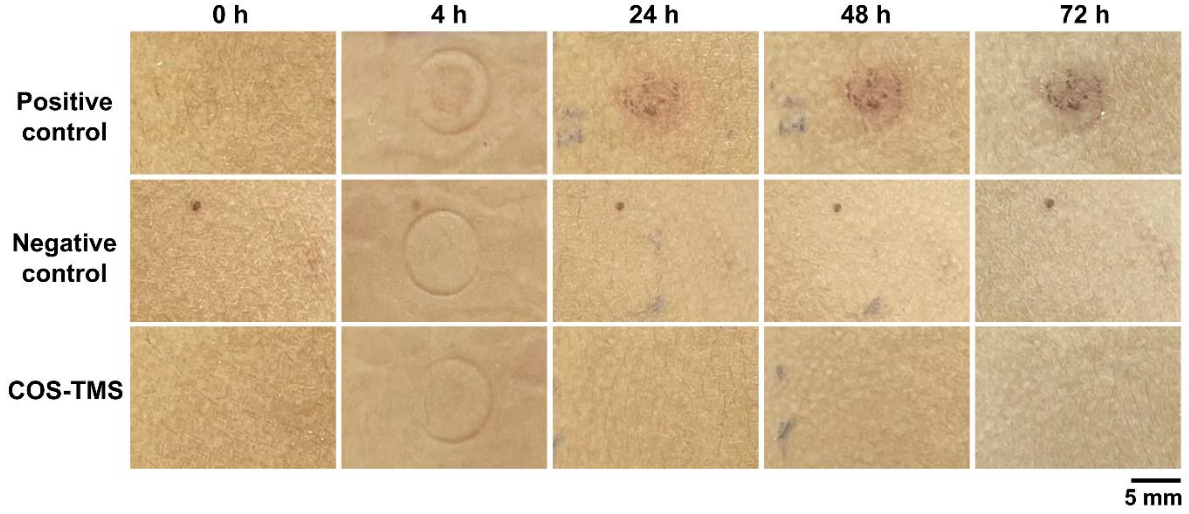

Figure S4. Visual appearance of human skin before (0 h) and after application of the positive control (20% w/w sodium lauryl sulfate solution), negative control (DI water), and cosmetic product in term of facial serum containing nanoemulsion with TMS (COS-TMS) for 4, 24, 48, and 72 h. Data are presented as mean ± SD from three independent experiments, n = 30.

The efficacy of the cosmetic product containing nanoemulsion with TMS was evaluated in 30 healthy volunteers who met the inclusion criteria and exhibited no signs of irritation in the preceding skin irritation test. A non-randomized, half-side comparison design was employed, in which each participant applied two drops the cosmetic product to the inner left forearm (5 × 5 cm^2), while the inner right forearm served as the untreated control. Participants were instructed to spread the cosmetic product evenly over the designated area without additional massage, as its low viscosity and good spreadability ensured sufficient coverage. The study was conducted over a 4-week period, with evaluations performed at baseline (week 0), week 1, and week 4. Prior to each evaluation, participants were instructed to gently cleanse the test area on their forearms using a wet tissue wipe and undergo a 30-min acclimatization period in a temperature-controlled room maintained at 25 °C. The skin biophysical parameters, including transepidermal water loss (TEWL), skin moisture, skin color, skin elasticity, and surface morphology, were measured using standardized instruments, including Tewameter® (TM 300, Courage + Khazaka electronic GmbH, Cologne, Germany), Corneometer® (CM 825, Courage + Khazaka electronic GmbH, Cologne, Germany), Colorimeter® (CL 400, Courage + Khazaka electronic GmbH, Cologne, Germany), Cutometer® (MPA 580, Courage + Khazaka electronic GmbH, Cologne, Germany), and Visioscan® (SV 700, Courage + Khazaka electronic GmbH, Cologne, Germany), respectively. All measurements were performed in triplicate.

The clinical efficacy evaluation in 30 human volunteers is shown in Figure S5. The results demonstrate that the facial serum containing nanoemulsion with TMS significantly improved multiple skin parameters compared with the control group over a 28-day period. Relative skin moisture increased markedly, with more than a 1.5-fold improvement observed after only 7 days ($p < 0.001$), and this effect persisted through day 28 ($p < 0.001$). This enhanced hydration can be explained by the nanoemulsion delivery system, which is known to increase the surface area of oil droplets and improve penetration of active compounds into the stratum corneum, thereby reinforcing barrier function and enhancing water retention (Zhou et al., 2025). The *in vitro* results demonstrated that TMS potently inhibited hyaluronidase, which reduces the degradation of hyaluronic acid and hence preserved natural moisturizing factors (NMFs). These findings were consistent with the clinical observations that the volunteers treated with the nanoemulsion containing the TMS showed improved skin hydration and water retention. The preserved NMFs likely enhanced the skin's ability to retain moisture, while the reinforced barrier function contributed to the observed long-term hydration effects. Therefore, the *in vitro* enzyme inhibition provided a mechanistic explanation for the clinical improvements in skin hydration.

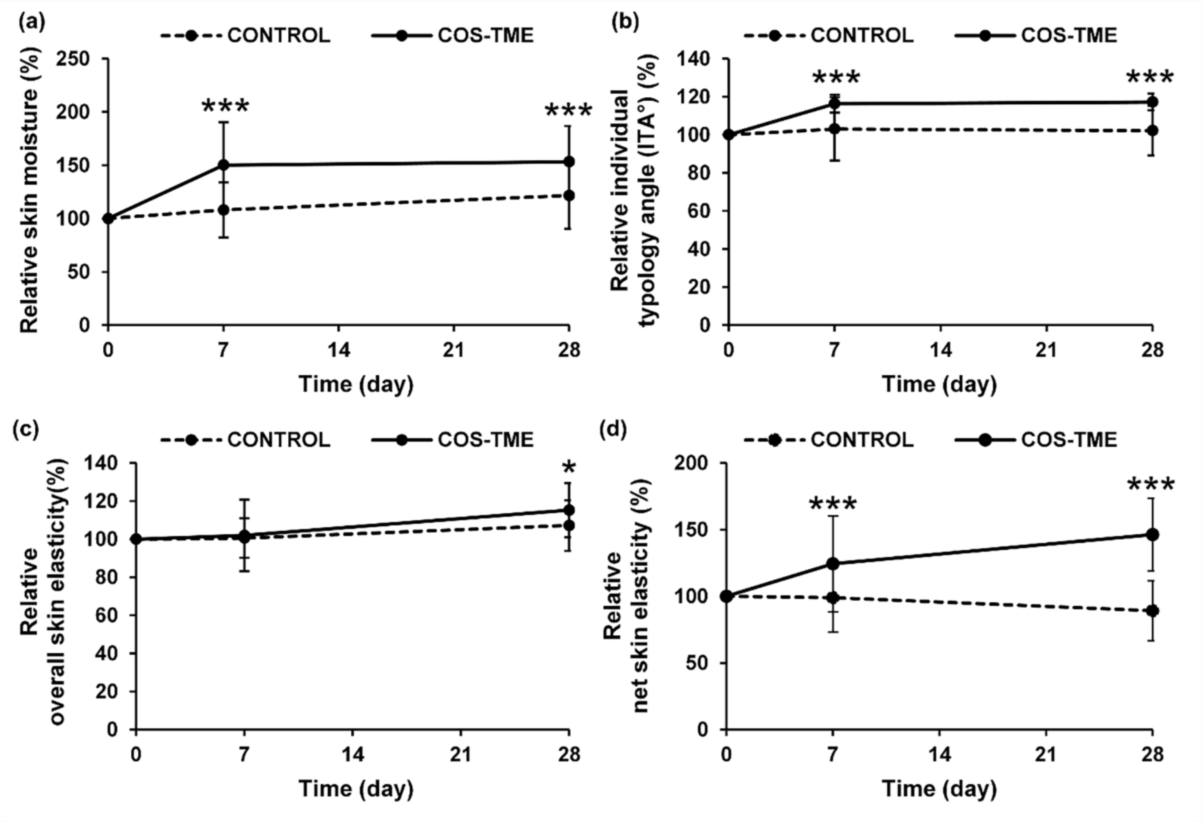

Figure S5. Relative skin moisture (a), individual typography angle (b), overall skin elasticity (c), and net skin elasticity (d) before and after application of the facial serum containing

nanoemulsion with TMS (COS-TMS) for 7 and 28 days, in a comparison with normal skin without any treatment (CONTROL). Data are presented as mean ± SD from three independent experiments, n = 30. Asterisks denote significant difference between COS-TMS and CONTROL (* $p < 0.05$ and *** $p < 0.001$).

In terms of skin brightness, the relative individual typology angle (ITA°) increased significantly after 7 days of treatment ($p < 0.001$) and remained elevated until day 28 ($p < 0.001$), suggesting that the facial serum containing nanoemulsion with TMS effectively promoted a visible brightening effect. This outcome aligned with the *in vitro* tyrosinase inhibition data, where TMS exhibited both the monophenolase and diphenolase inhibitory activity. Additionally, the incorporation into a nanoemulsion delivery system likely improved skin penetration and sustained release, enhancing the bioavailability of active compounds at the epidermal level (Harwansh et al., 2019). Consequently, the combined effect of enzyme inhibition and efficient delivery can explain the observed improvement in skin brightness over the treatment period.

Improvements in skin elasticity were also evident, with overall elasticity showing a gradual increase that became statistically significant after 28 days ($p < 0.05$). This delayed but consistent effect suggested that prolonged application supported skin hydration and barrier function, likely through inhibition of hyaluronidase by TMS. However, more immediate changes were observed in relative net skin elasticity, which increased significantly after just 7 days ($p < 0.001$) and remained improved throughout the study period ($p < 0.001$), reflecting enhanced dermal resilience and firmness. By reducing the degradation of hyaluronic acid, the TMS may help preserve NMFs, thereby enhancing water retention and potentially contributing to wrinkle reduction and maintenance of skin elasticity (Chylinska and Maciejczyk, 2025).

The combination of rapid improvements in hydration, brightness, and net elasticity with the slower but steady enhancement of overall elasticity highlights both the short-term and long-term benefits of facial serum containing nanoemulsion with TMS. The results confirm that the facial serum provided improvements in hydration, brightening, and elasticity, supporting its potential application as an innovative multifunctional cosmeceutical product targeting moisturization, skin tone enhancement, and anti-aging benefits.

References

Azeem, A., Rizwan, M., Ahmad, F.J., Iqbal, Z., Khar, R.K., Aqil, M., Talegaonkar, S., 2009. Nanoemulsion components screening and selection: a technical note. AAPS PharmSciTech 10(1), 69–76. https://doi.org/10.1208/s12249-008-9178-x

Baptista, S., Baptista, F., Freitas, F., 2023. Development of emulsions containing L-ascorbic acid and α-tocopherol based on the polysaccharide FucoPol: stability evaluation and rheological and texture assessment. Cosmetics 10(2), 56. https://doi.org/10.3390/cosmetics10020056

Basketter, D.A., York, M., McFadden, J.P., Robinson, M.K., 2004. Determination of skin irritation potential in the human 4-h patch test. Contact Dermatitis 51(1), 1–4. https://doi.org/10.1111/j.0105-1873.2004.00385.x

Cevikkalp, S.A., Loker, G.B., Yaman, M., Amoutzopoulos, B., 2016. A simplified HPLC method for determination of tryptophan in some cereals and legumes. Food Chem. 193, 26–29. https://doi.org/10.1016/j.foodchem.2015.02.108

Chaiyana, W., Jiamphun, S., Bezuidenhout, S., Yeerong, K., Krueathanasing, N., Thammasorn, P., Jittasai, P., Tanakitvanicharoen, S., Tima, S., Anuchapreeda, S., 2023. Enhanced cosmeceutical potentials of the oil from *Gryllus bimaculatus* de Geer by nanoemulsions. Int. J. Nanomed. 18, 2955–2972. https://doi.org/10.2147/IJN.S406864

Choi, B.D., Wong, N.A., Auh, J.H., 2017. Defatting and sonication enhances protein extraction from edible insects. Korean J. Food Sci. Anim. Resour. 37(6), 955. https://doi.org/10.5851/kosfa.2017.37.6.955

Chylinska, N., Maciejczyk, M., 2025. Hyaluronic acid and skin: its role in aging and wound-healing processes. Gels 11(4), 281. https://doi.org/10.3390/gels11040281

Couteau, C., Coiffard, L., 2016. Overview of skin whitening agents: Drugs and cosmetic products. Cosmetics 3(3), 27. https://doi.org/10.3390/cosmetics3030027

Cunniff, P., Washington, D., 1997. Official methods of analysis of AOAC International. J. AOAC Int. 80(6), 127A. https://doi.org/10.1093/jaoac/80.6.127A

Duracher, L., Visdal-Johnsen, L., Mavon, A., 2015. In vitro and in vivo dermal absorption assessment of acetyl aspartic acid: A compartmental study. Int. J. Cosmet. Sci. 37(1), 34–40. https://doi.org/10.1111/ics.12255

Fernandez-Julia, P.J., Tudela-Serrano, J., Garcia-Molina, F., Garcia-Canovas, F., Garcia-Jimenez, A., Munoz-Munoz, J.L., 2021. Study of tyrosine and dopa enantiomers as tyrosinase substrates initiating L- and D-melanogenesis pathways. Biotechnol. Appl. Biochem. 68(4), 823–831. https://doi.org/10.1002/bab.1998

Fuziwara, S., Inoue, K., Denda, M., 2003. NMDA-type glutamate receptor is associated with cutaneous barrier homeostasis. J. Invest. Dermatol. 120(6), 1023–1029. https://doi.org/10.1046/j.1523-1747.2003.12238.x

Ganguly, K., Dutta, S.D., Jeong, M.S., Patel, D.K., Cho, S.J., Lim, K.T., 2021. Naturally-derived protein extract from *Gryllus bimaculatus* improves antioxidant properties and promotes osteogenic differentiation of hBMSCs. PLoS One 16(6), e0249291. https://doi.org/10.1371/journal.pone.0249291

Gasmi, A., Mujawdiya, P.K., Lysiuk, R., Shanaida, M., Peana, M., Piscopo, S., Beley, N., Dzyha, S., Smetanina, K., Shanaida, V., Resimont, S., Bjorklund, G., 2025. The possible roles of β-alanine and L-carnosine in anti-aging. Curr. Med. Chem. 32(1), 6–22. https://doi.org/10.2174/0109298673263561231117054447

Ghosh, S., Lee, S.M., Jung, C., Meyer-Rochow, V.B., 2017. Nutritional composition of five commercial edible insects in South Korea. J. Asia-Pac. Entomol. 20(2), 686–694. https://doi.org/10.1016/j.aspen.2017.04.003

Gillbro, J.M., Merinville, E., Cattley, K., Al-Bader, T., Hagforsen, E., Nilsson, M., Mavon, A., 2015. In vivo topical application of acetyl aspartic acid increases fibrillin-1 and collagen IV deposition leading to a significant improvement of skin firmness. Int. J. Cosmet. Sci. 37(1), 41–46. https://doi.org/10.1111/ics.12255

Harwansh, R.K., Deshmukh, R., Rahman, M.A., 2019. Nanoemulsion: promising nanocarrier system for delivery of herbal bioactives. J. Drug Deliv. Sci. Technol. 51, 224–233. https://doi.org/10.1016/j.jddst.2019.03.006

Hou, Y., Wu, G., 2017. Nutritionally nonessential amino acids: A misnomer in nutritional sciences. Adv. Nutr. 8(1), 137. https://doi.org/10.3945/an.116.012971

Hou, Y., Yin, Y., Wu, G., 2015. Dietary essentiality of "nutritionally non-essential amino acids" for animals and humans. Exp. Biol. Med. (Maywood) 240(8), 997–1007. https://doi.org/10.1177/1535370215587913

Jara, C.P., de Andrade Berti, B., Mendes, N.F., Engel, D.F., Zanesco, A.M., Pereira de Souza, G.F., Bezerra, R.M., Bagatin, J.T., Maria-Engler, S.S., Morari, J., Velander, W.H., Velloso, L.A., Araujo, E.P., 2021. Glutamic acid promotes hair growth in mice. Sci. Rep. 11(1), 15453. https://doi.org/10.1038/s41598-021-94816-y

Kowalska, M., Turek, P., Zbikowska, A., Babut, M., Szakiel, J., 2021. The quality of emulsions with new synthesized lipids stabilized by xanthan gum. Biomolecules 11(2), 213. https://doi.org/10.3390/biom11020213

Kuo, C., Fisher, B.L., 2022. A literature review of the use of weeds and agricultural and food industry by-products to feed farmed crickets (Insecta; Orthoptera; Gryllidae). Front. Sustain. Food Syst. 5, 810421. https://doi.org/10.3389/fsufs.2021.810421

Lovrien, R., Matulis, D., 1995. Assays for total protein. Curr. Protoc. Protein Sci. 1(1), 3–4. https://doi.org/10.1002/0471140864.ps0304s01

Ma, X., Wang, H., Song, Y., Pan, Y., 2021. Skin irritation potential of cosmetic preservatives: An exposure-relevant study. J. Cosmet. Dermatol. 20(1), 195–203. https://doi.org/10.1111/jocd.13502

Magara, H.J., Niassy, S., Ayieko, M.A., Mukundamago, M., Egonyu, J.P., Tanga, C.M., Fiaboe, K.K.M., Hugel, S., Orinda, M.A., Roos, N., Ekesi, S., 2020. Edible crickets (Orthoptera) around the world: Distribution, nutritional value, and other benefits—a review. Front. Nutr. 7, 537915. https://doi.org/10.3389/fnut.2020.537915

Magrode, N., Poomanee, W., Kiattisin, K., Ampasavate, C., 2024. Microemulsions and nanoemulsions for topical delivery of tripeptide-3. Pharmaceutics 16(4), 554. https://doi.org/10.3390/pharmaceutics16040554

Manap, A.S.A., Lum, Y.K., Ong, L.H., Tang, Y.Q., Gew, L.T., Chia, A.Y.Y., 2021. Perspective approaches on melanogenesis inhibition. Dermatol. Sin. 39(1), 1–12. https://doi.org/10.4103/ds.ds_46_20

Masoongnoen, J., Sansenya, S., Wechakorn, K., 2023. GC–MS profiling and bioactivity of edible-cricket extracts. Agric. Nat. Resour. 57(6), 917–932. https://doi.org/10.34044/j.anres.2023.57.6.02

Mba, A.R.F., David-Briand, E., Viau, M., Riaublanc, A., Kansci, G., Genot, C., 2021. Protein extraction yield and emulsifying properties of *Rhynchophorus phoenicis* larvae. Future Foods 4, 100037. https://doi.org/10.1016/j.fufo.2021.100037

Mohiuddin, A.K., 2019. Skin aging & modern anti-aging strategies. Int. J. Clin. Dermatol. Res. 7, 209–240. https://doi.org/10.19070/2332-2977-1900052

Nagai, K., Suda, T., Kawasaki, K., Mathuura, S., 1986. Action of carnosine and β-alanine on wound healing. Surgery 100(5), 815–821.

Orkusz, A., Dyminska, L., Banas, K., Harasym, J., 2024. Chemical and nutritional fat profile of edible insects. Foods 13(1), 32. https://doi.org/10.3390/foods13010032

Papakonstantinou, E., Roth, M., Karakiulakis, G., 2012. Hyaluronic acid: A key molecule in skin aging. Dermatoendocrinol. 4(3), 253–258. https://doi.org/10.4161/derm.21923

Perera, G.C., Bhujel, R.C., 2022. Replacement of fishmeal by house cricket (*Acheta domesticus*) and field cricket (*Gryllus bimaculatus*) meals: Effect on growth,

pigmentation, and breeding performance of guppy (*Poecilia reticulata*). Aquac. Rep. 25, 101260. https://doi.org/10.1016/j.aqrep.2022.101260

Perera, G.C., Senanayake, S.N., Sandaruwani, D.R., Salgadu, M.K.S., Rajapakshe, A.D.W.R., Athauda, S., 2025. Replacement of fishmeal by three cricket meals (*Acheta domesticus*, *Gryllus bimaculatus, Teleogryllus mitratus*) in swordtail (*Xiphophorus helleri*) fry feed: Effect on growth, stress tolerance, pigmentation and histopathological alterations. Turk. J. Fish. Aquat. Sci. 25(7), TRJFAS26460. https://doi.org/10.4194/TRJFAS26460

Proksch, E., 2018. pH in nature, humans and skin. J. Dermatol. 45(9), 1044–1052. https://doi.org/10.1111/1346-8138.14489

Quinteros, M.F., Martinez, J., Barrionuevo, A., Rojas, M., Carrillo, W., 2022. Functional, antioxidant, and anti-inflammatory properties of cricket protein concentrate (*Gryllus assimilis*). Biology 11(5), 776. https://doi.org/10.3390/biology11050776

Romanowski, P., Schueller, R., 2001. Stability testing of cosmetic products. In: Handbook of Cosmetic Science and Technology. CRC Press, Boca Raton, pp. 785–796

Serre, C., Busuttil, V., Botto, J.M., 2018. Intrinsic and extrinsic regulation of human skin melanogenesis and pigmentation. Int. J. Cosmet. Sci. 40(4), 328–347. https://doi.org/10.1111/ics.12466

Singh, B.K., Park, S.H., Lee, H.B., Goo, Y.A., Kim, H.S., Cho, S.H., Lee, J.H., Ahn, G.W., Kim, J.P., Kang, S.M., Kim, E.K., 2016. Kojic acid peptide: A new compound with anti-tyrosinase potential. Ann. Dermatol. 28(5), 555–561. https://doi.org/10.5021/ad.2016.28.5.555

Solano, F., 2014. Melanins: Skin pigments and much more—types, structural models, biological functions, and formation routes. New J. Sci. 2014(1), 498276. https://doi.org/10.1155/2014/498276

Solano, F., 2020. Metabolism and functions of amino acids in the skin. In: Wu, G. (Ed.), Amino Acids in Nutrition and Health. Adv. Exp. Med. Biol., Springer, London, pp. 187–199.

Steiling, W., Bracher, M., Courtellemont, P., De Silva, O., 1999. The HET–CAM, a useful in vitro assay for assessing the eye irritation properties of cosmetic formulations and ingredients. Toxicol. In Vitro 13(2), 375–384. https://doi.org/10.1016/S0887-2333(98)00091-5

Udomsil, N., Imsoonthornruksa, S., Gosalawit, C., Ketudat-Cairns, M., 2019. Nutritional values and functional properties of house cricket (*Acheta domesticus*) and field cricket (*Gryllus bimaculatus*). Food Sci. Technol. Res. 25(4), 597–605. https://doi.org/10.3136/fstr.25.597

Yeerong, K., Chantawannakul, P., Anuchapreeda, S., Rades, T., Mullertz, A., Chaiyana, W., 2024a. Acheta domesticus: A natural source of anti-skin-aging ingredients for cosmetic applications. Pharmaceuticals 17(3), 346. https://doi.org/10.3390/ph17030346

Yeerong, K., Chantawannakul, P., Anuchapreeda, S., Wangtueai, S., Chaiyana, W., 2024b. Optimization of hydrolysis conditions, isolation, and identification of biologically active peptides derived from *Acheta domesticus* for antioxidant and collagenase inhibition. Antioxidants 13(3), 367. https://doi.org/10.3390/antiox13030367

Zhang, X.J., Chinkes, D.L., Wolfe, R.R., 2004. Leucine supplementation has an anabolic effect on proteins in rabbit skin wound and muscle. J. Nutr. 134(12), 3313–3318.

Zhou, Y., Wu, L., Zhang, Y., Hu, J., Fardous, J., Ikegami, Y., Ijima, H., 2025. Topical delivery of ceramide by oil-in-water nanoemulsion to retain epidermal moisture content in dermatitis. Biomolecules 15(5), 608. https://doi.org/10.3390/biom15050608
